# Prospective proof-of-concept observational RESEarch about traditional herbal preparation Treatment for Chronic Obstructive Pulmonary Disease (RESET-COPD-1)

**DOI:** 10.3389/fphar.2024.1437253

**Published:** 2024-09-26

**Authors:** Dasol Park, Jungtae Leem, Beom-Joon Lee, Kwan-Il Kim, Hee-Jae Jung

**Affiliations:** ^1^ Department of Diagnostics, College of Korean Medicine, Wonkwang University, Iksan, Republic of Korea; ^2^ Korean Medicine Clinical Research Institute, Wonkwang University Korean Medicine Hospital, Iksan, Republic of Korea; ^3^ Research Center of Traditional Korean Medicine, College of Korean Medicine, Wonkwang University, Iksan, Republic of Korea; ^4^ Department of Clinical Korean Medicine, College of Korean Medicine, Graduate School, Kyung Hee University, Seoul, Republic of Korea; ^5^ Division of Allergy, Immune and Respiratory System, Department of Internal Medicine, College of Korean Medicine, Kyung Hee University, Kyung Hee University Medical Center, Seoul, Republic of Korea

**Keywords:** cheongsangboha-tang, chronic obstructive pulmonary disease, dyspnea, herbal preparation, prospective observational study, respiratory disease, Qing Shang Bu Xia Tang, proof-of-concept study

## Abstract

**Background:**

Despite advances in medical science, chronic obstructive pulmonary disease (COPD) continues to impact patients’ lives significantly, due to symptom management limitations. Cheongsangboha-tang (CSBHT; Qing Shang Bu Xia Tang) and Hyunggaeyeongyo-tang (HGYGT; Jing Jie Lian Qiao Tang) have been used to treat respiratory diseases, including COPD. However, clinical data supporting their efficacy are lacking. We prospectively observed the response of patients with COPD to CSBHT and HGYGT as adjunctive therapies and assessed the feasibility of future research.

**Methods:**

Patients with COPD who were prescribed adjunctive HGYGT or CSBHT according to the COPD clinical practice protocol of Kyung Hee University Korean Medicine Hospital were recruited. Participants visited the hospital every month, for 6 months, to receive herbal preparations according to a Korean Medicine doctor’s diagnosis and prescription and outcome evaluations. The primary outcome was the 6-min walking test (6-MWT). Secondary outcomes included the pulmonary function test (PFT), COPD Assessment Test (CAT), St. George’s Respiratory Questionnaire (SGRQ), and modified Medical Research Council (mMRC) score. Syndrome differentiation, adverse events, and patient adherence were recorded.

**Results:**

Thirty-seven patients were initially enrolled and followed up for a mean period of 154.1 days. CSBHT was prescribed to 36 patients, while one patient received either CSBHT or HGYGT, or both, throughout the entire period. During the herbal preparation treatment period, no statistically significant changes were observed in the 6-MWT. The CAT score (mean ± standard deviation) changed from 17.0 ± 5.0 to 12.5 ± 3.6, and the visual analogue scale score for dyspnea changed from 47.5 ± 18.9 to 28.4 ± 18.6 (both statistically significant from visit 5). The coronavirus disease 2019 pandemic precluded the PFT. SGRQ and mMRC scores did not change significantly. During the study period, seven patients dropped out, two experienced mild dyspepsia, and one experienced mild headache. No serious adverse effects were observed.

**Conclusion:**

We illustrated the therapeutic potential of CSBHT and provided preliminary clinical data on its efficacy and safety in patients with COPD. Our study highlights the need to derive optimal herbal formulations, which should be administered for an appropriate duration, based on the therapeutic goals for the treatment of COPD.

## 1 Introduction

Chronic obstructive pulmonary disease (COPD) is characterized by persistent respiratory symptoms and airflow obstruction (Global Initiative for Chronic Obstructive Lung Disease, 2023). COPD patients exhibit abnormal inflammatory responses to inhaled toxic particles and gases. These responses recurrently affects the bronchi, bronchioles, or alveoli in genetically predisposed individuals, resulting in progressive damage to the airways, lung parenchyma, and vascular structures (Hogg and Timens, 2009). Major clinical features of COPD, such as dyspnea and cough, and the vulnerability to exacerbations cause substantial limitations in daily activities, as well as significant morbidity (Miravitlles and Ribera, 2017). Additionally, patients with COPD often present with multiple comorbidities, including diseases such as cardiovascular diseases, lung cancer, anxiety, or depression, influencing the prognosis and overall condition of affected individuals (Cavaillès et al., 2013).

COPD has a high prevalence globally. A study published in 2015 reported an increase in the prevalence of COPD in individuals aged 30 and over, from 10.7% in 1990 to 384 million cases by 2010, marking a 68.9% surge (Adeloye et al., 2015). In 2015, COPD was the third leading cause of death worldwide, claiming the lives of 3.2 million patients annually (Wang et al., 2016). In 2016, the 1-year mortality rate was reported as 21% and the 5-year mortality rate as 55%, with annual direct costs of $18 billion in the United States alone (López-Campos et al., 2016). A population-based survey conducted across 12 countries, also published in 2016, indicated that the indirect costs of COPD, because of work limitations due to COPD symptoms or related multiple comorbidities, significantly exceeded the direct costs (Foo et al., 2016; Iheanacho et al., 2020).

Bronchodilators, such as beta-2 agonists and anticholinergics, used to attenuate airflow limitation in stable conditions, anti-inflammatory agents, including inhaled corticosteroids, and antibiotics, for preventing and managing exacerbations, are the standard pharmacotherapies for COPD (Global Initiative for Chronic Obstructive Lung Disease, 2023). However, these therapies have some limitations that need to be addressed. Current pharmacotherapy has been shown to be effective for symptomatic relief in COPD patients, but disease-modifying therapy has not yet been identified (Brandsma et al., 2020). Moreover, pharmacological treatments alone often fall short in improving symptoms. Therefore, the GOLD guidelines recommend a multifaceted treatment approach (Global Initiative for Chronic Obstructive Lung Disease, 2023). The therapeutic goals for COPD include alleviating symptoms, enhancing exercise capacity, and improving quality of life. Consequently, interest in treatments other than standard pharmacotherapy that can improve the quality of life through symptom management without adverse effects is currently increasing.

Traditional herbal preparations have been clinically utilized for COPD treatment and management (Rahman et al., 2022). The number of randomized controlled trials (RCTs) validating the effectiveness and safety of traditional herbal preparation for COPD is increasing, with recent experimental studies confirming their therapeutic potential by exploring active compounds and molecular mechanisms (Feng et al., 2022; Cao et al., 2023). At Kyung Hee University Korean Medicine Hospital (KHMH), alongside standard pharmacotherapy, patients with COPD are treated with Cheongsangboha-tang (CSBHT; Qing Shang Bu Xia Tang) and Hyunggaeyeongyo-tang (HGYGT; Jing Jie Lian Qiao Tang), based on clinical symptoms, co-occurrence of conditions, such as rhinitis or asthma, and syndrome differentiation.

Modified CSBHT has been shown to attenuate the hazardous effects of lung inflammation in COPD-like mouse models, indicating its potential use in COPD (Lee H. et al., 2012; Jung et al., 2013). Furthermore, retrospective studies administering CSBHT to patients with chronic respiratory diseases have noted improvements in lung function as well as significant reductions in IgE levels (Bang et al., 2011; Baek et al., 2016a). Retrospective studies on modified HGYGT in patients presenting with cough as their chief complaint have demonstrated improvements in the clinical symptom scores for cough and sputum (Baek et al., 2016b).

Further investigation into the effects of CSBHT and HGYGT on COPD is necessary. However, clinical data regarding appropriate dosage, treatment duration, effectiveness, concomitant medication, adherence, and side effects are currently insufficient but essential for developing protocols for controlled clinical trials. A single-arm, uncontrolled prospective observational study can provide data on safety, efficacy, and future controlled study design (Wang et al., 2024). Therefore, this prospective observational study was conducted in patients with COPD undergoing standard pharmacotherapy in conjunction with CSBHT or HGYGT, to evaluate the clinical response to and safety of these adjunctive herbal preparations, gather basic data, and assess the feasibility of future research protocols.

## 2 Methods

### 2.1 Study design and approval

#### 2.1.1 Study design

We conducted a 6-month, prospective, observational, proof-of-concept study on patients with COPD who had already received standard medical treatment at KHMH and were prescribed adjunctive HGYGT or CSBHT according to the KHMH COPD clinical practice protocol. This study aimed to evaluate the effectiveness (clinical response), safety, adherence, and dropout rates. The patient visits and evaluations followed the KHMH clinical practice protocol. Patients for whom HGYGT or CSBHT was not indicated were prescribed alternative herbal preparations and were excluded from this study.

#### 2.1.2 Ethics approval and protocol registration

The study protocol was approved by the institutional review board of KHMH (approval no.: KOMCIRB-2020–12–005–002; approval date: 30 March 2021) and was registered at cris.nih.go.kr (KCT0006716). Written informed consent was obtained from all participants in accordance with the tenets of the Declaration of Helsinki.

### 2.2 Participants

#### 2.2.1 Participant recruitment

Participants for this observational study were recruited from among patients with COPD who visited the outpatient clinic at KHMH and who commenced herbal treatment after the study began.

#### 2.2.2 Eligibility criteria

Participants who met the following criteria were included: Adults aged 40–80 years; prescription of HGYGT or CSBHT for treating COPD at KHMH; meeting the clinical diagnostic criteria for COPD (FEV_1_/FVC <0.70 on spirometry according to the Global Initiative for Chronic Obstructive Lung Disease [GOLD] standard (Global Initiative for Chronic Obstructive Lung Disease - GOLD, 2020)); voluntary written consent to participate in the study.

We excluded the following individuals: Participants for whom herbal preparation treatment was deemed not indicated based on the attending physician’s clinical judgement; those who had received Korean medical treatment for respiratory diseases at another Korean medical institution within the last 30 days; pregnant or nursing women.

Patients’ participation was also terminated in the following cases: If a participant withdrew consent for participation; if a participant did not meet the inclusion criteria or met the exclusion criteria; if the patient did not receive HGYGT or CSBHT for a period exceeding 6 months, even if written consent was provided and eligibility criteria were met; and if the patient refused to participate during the course of the study.

#### 2.2.3 Sample size calculation

This was an observational study designed to determine the sample size for a subsequent RCT, verify feasibility, and gather information on the regimen required for observational research; therefore, sample size calculation was not necessary. However, based on research that suggested a minimum of 12 participants per group for preliminary clinical trials, we planned to recruit more than 12 participants (Julious, 2005). Taking into account a minimum follow-up (f/u) period of 6-months and the monthly number of newly registered COPD patients at KHMH, we calculated the maximum number of study subjects to be 30.

### 2.3 Interventions

#### 2.3.1 Standard medical treatment

Participants were treated with standard medical treatment according to the COPD Clinical Practice Guidelines of the Korean Academy of Tuberculosis and Respiratory Diseases, revised in 2018 (Park et al., 2018). Changes in treatment were monitored and recorded monthly in case-report forms. All other medications were recorded as concomitantly used drugs.

#### 2.3.2 Adjuvant herbal preparation treatment

The formulations for HGYGT and CSBHT are presented in Table 1. The names of the botanical drugs in HGYGT and CSBHT were denoted by scientific names based on the Medicinal Plant Names Services database (https://mpns.science.kew.org/mpns-portal/) as of 1 August 2024. Licensed Korean Medicine practitioners prescribed and administered HGYGT and CSBHT to the participants.

**TABLE 1:** Botanical drugs in Hyunggaeyeongyo-tang (HGYGT) and Cheongsangboha-tang (CSBHT) and their doses for single administration.

Scientific name with botanical drug name	Chinese name	Pinyin name	Single dose (g)
			Granule
Hyunggaeyeongyo-tang (HGYGT; Jing Jie Lian Qiao tang)			
*Nepeta tenuifolia* Benth. [Lamiaceae; Schizonepetae Spica]	荊芥	Jin Jie	0.172
*Forsythia viridissima* Lindl. [Oleaceae; Forsythiae Fructus]	連翹	Lian Qiao	0.342
*Saposhnikovia divaricate* (Turcz. ex Ledeb.) Schischk. [Apiaceae; Saposhnikoviae Radix]	防風	Fang Feng	0.385
*Angelica gigas* Nakai [Apiaceae; Angelicae Gigantis Radix]	當歸	Dang Gui	0.315
*Ligusticum officinale* (Makino) Kitag. [Apiaceae; Cnidii Rhizoma]	川芎	Chuan Xiong	0.307
*Paeonia lactiflora* Pall. [Paeoniaceae; Paeoniae Radix]	芍藥	Shao Yao	0.326
*Bupleurum falcatum* L. [Apiaceae; Bupleuri Radix]	柴胡	Chai Hu	0.176
*Citrus x aurantium* L. [Rutaceae; Aurantii Fructus Immaturus]	枳殼	Zhi Qiao	0.525
*Scutellaria baicalensis* Georgi [Lamiaceae; Scutellariae Radix]	黃芩	Huang Qin	0.395
*Gargenia jasminoides* J. Ellis [Rubiaceae; Gardeniae Fructus]	梔子	Zhi Zi	0.414
*Angelica dahurica* (Hoffm.) Benth. and Hook.f. ex Franch. and Sav. [Apiaceae; Angelicae Dahuricae Radix]	白芷	Bai Zhi	0.295
*Platycodon grandiflorus* (Jacq.) A.DC. [Campanulaceae; Platycodonis Radix]	桔梗	Jie Geng	0.446
*Glycyrrhiza uralensis* Fisch. ex DC. [Fabaceae; Glycyrrhizae Radix et Rhizoma]	甘草	Gan Cao	0.203
Cheongsangboha-tang (CSBHT; Qing Shang Bu Xia Tang)			
*Rehmannia glutinosa* (Gaertn.) DC. [Orobanchaceae; Rehmanniae Radix Preparata]	熟地黃	Shu Di Huang	0.98
*Dioscorea polystachya* Turcz. [Dioscoreaceae; Dioscoreae Rhizoma]	山藥	Shan Yao	0.73
*Cornus officinalis* Sieblod and Zucc. [Cornaceae; Corni Fructus]	山茱萸	Shan Zhu Yu	0.73
*Paeonia x suffruticosa* Andrews [Paeoniaceae; Moutan Radicis Cortex]	牧丹皮	Mu Dan Pi	0.49
*Poria cocos* (Schw.) Wolf [Polyporaceae; Poria Sclerotium]	茯苓	Fu Ling	0.49
*Alisma plantago-aquatica subsp. orientale* (Sam.) Sam. [Alismataceae; Alismatis Rhizoma]	澤瀉	Ze Xie	0.49
*Citrus trifoliata* L. [Rutaceae; Ponciri Fructus Immaturus]	枳實	Zhi Shi	0.37
*Coptis japonica* (Thunb.) Makino [Ranunculaceae; Coptidis Rhizoma]	黃連	Huang Lian	0.37
*Trichosanthes kirilowii* Maxim. [Cucurbitaceae; Trichosanthis Semen]	瓜樓仁	Gua Lou Zi	0.37
*Scutellaria baicalensis* Georgi [Lamiaceae; Scutellariae Radix]	黃芩	Huang Qin	0.37
*Schisandra chinensis* (Turcz.) Baill. [Schisandraceae; Schisandrae Fructus]	五味子	Wu Wei Zi	0.37
*Liriope muscari* (Decne.) L.H.Bailey [Asparagaceae; Liriopis seu Ophiopogonis Tuber]	麥門冬	Mai Dong	0.37
*Asparagus cochinchinensis* (Lour.) Merr. [Asparagaceae; Asparagi Tuber]	天門冬	Tian Dong	0.37
*Fritillaria thunbergia* Miq. [Liliaceae, Fritillariae Thunbergii Bulbus]	折貝母	Zhe Bei Mu	0.37
*Platycodon grandiflorus* (Jacq.) A.DC. [Campanulaceae; Platycodonis Radix]	桔梗	Jie Geng	0.37
*Prunus armeniaca* L. [Rosaceae; Armeniacae Semen]	杏仁	Ku Xing Ren	0.37
*Pinellia ternate* (Thunb.) Makino [Araceae; Pinelliae Tuber]	半夏	Ban Xia	0.37
*Glycyrrhiza uralensis* Fisch. ex DC. [Fabaceae; Glycyrrhizae Radix et Rhizoma]	甘草	Gan Cao	0.24

We used HGYGT in the form of an extract powder manufactured according to the herbal standards and quality control guidelines for herbal preparations in The Korean Herbal Pharmacopoeia by Hankookshinyak Corp. in South Korea and marketed as an over-the-counter drug (Korea Drug Code 200002258) (Ministry of Food and Drug Safety, 2024). CSBHT was an extract powder prepared by Kyung Hee University Korean Medicine Hospital, following the standards of the Korean Herbal Pharmacopoeia for herbal drug specifications and the manufacturing and quality control guidelines for herbal (botanical) preparations. CSBHT was prepared as follows: Extraction: The botanical drugs were combined in the following specified ratios; Rehmanniae Radix Preparata (8,800 g), Dioscoreae Rhizoma (6,600 g), Corni Fructus (6,600 g), Moutan Radicis Cortex (4,400 g), Poria Sclerotium (4,400 g), Alismatis Rhizoma (4,400 g), Ponciri Fructus Immaturus (3,300 g), Coptidis Rhizoma (3,300 g), Trichosanthis Semen (3,300 g), Scutellariae Radix (3,300 g), Schisandrae Fructus (3,300 g), Liriopis seu Ophiopogonis Tuber (3,300 g), Asparagi Tuber (3,300 g), Fritillariae Thunbergii Bulbus (3,300 g), Platycodonis Radix (3,300 g), Armeniacae Semen (3,300 g), Pinelliae Tuber (3,300 g), Glycyrrhizae Radix et Rhizoma (2,200 g). These were placed in an extraction tank with approximately 500 L of purified water, heated to 80°C–90°C, and maintained at this temperature for 90 min; Filtration: Immediately after extraction, a high-speed centrifugal filter (20,000–30,000 rpm) was used to separate solids from the liquid; Concentration: Excipients (3500 g dextrin, 2000g lactose) were dissolved in the filtered extract, and then concentrated at a low temperature (56°C–60°C) using a thin film evaporator under reduced pressure; Spray drying: The concentrated solution was dried using a spray dryer; Granulation: A binder [10% Povidone (PVP, Kollidon^®^30) in 90% ethanol] was added to the dried extract and granulated using a wet granulator; Sieving: The granules were sieved using a 1.7mm mesh, then centrifuged to obtain the final granules; Packaging: An automatic packaging machine was used to pack 3 g per packet, producing approximately 9,000 packets. All botanical drugs used in the study complied with CITES regulations and underwent quality inspections, including sensory, hazardous substance, and precision testing by the Ministry of Food and Drug Safety of South Korea, ensuring their import and distribution quality. The formulation used in this study was based on traditional knowledge disseminated throughout East Asia centuries ago and is not subject to the Nagoya Protocol.

#### 2.3.3 Concomitant Korean medical treatments

Patients taking other herbal preparations or undergoing other Korean medical treatments (acupuncture, moxibustion, etc.) for respiratory diseases met the exclusion criteria. However, because this was an observational study, the use of other Korean or conventional medical treatments initiated after the start of the study was not restricted, and any such treatments were recorded in the case report forms every month.

### 2.4 Clinical assessment

The detailed schedule of the study is presented in Table 2.

**TABLE 2:** Schedule of the study.

Time point (month)	Visit 1 (baseline)	Visit 2 (1 m)	Visit 3 (2 m)	Visit 4 (3 m)	Visit 5 (4 m)	Visit 6 (5 m)	Visit 7 (6 m)
Month	0	1	2	3	4	5	6
Visit window (day)		0	±3	±3	±3	±3	±3
Enrollment							
Eligibility screening	●						
Written informed consent	●						
Vital signs and physical examination	●	●	●	●	●	●	●
Sociodemographic characteristics	●						
Medical history	●	●	●	●	●	●	●
Alcohol and smoking history	●						
Visit scheduling education	●	●	●	●	●	●	●
Intervention							
Herbal prescription	●	●	●	●	●	●	
Assessment							
6-MWT	●			●			●
PFT	●			●			●
CAT	●	●	●	●	●	●	●
SGRQ	●	●	●	●	●	●	●
mMRC	●	●	●	●	●	●	●
VAS for dyspnea	●	●	●	●	●	●	●
Syndrome Differentiation Assessment for COPD	●						●
Changes in symptoms (the frequency of COPD exacerbations)	●	●	●	●	●	●	●
Changes in medical history, medication, or treatments		●	●	●	●	●	●
Hematologic tests	●			●			●
Adverse events		●	●	●	●	●	●
Drop-out rate and compliance		●	●	●	●	●	●

Hematological tests: CRP, CBC, ALP, AST, ALT, total bilirubin, direct bilirubin; BUN, and Cr.

6-MWT, 6-min walking test; ALP, alkaline phosphatase; ALT, alanine transaminase; AST, aspartate aminotransferase; BUN, blood urea nitrogen; CAT, chronic obstructive pulmonary disease assessment test; CBC, complete blood count; COPD, chronic obstructive pulmonary disease; Cr, creatinine; CRP, C-reactive protein; GGT, gamma-glutamyl transferase; mMRC, modified Medical Research Council; PFT, pulmonary function test; SGRQ, St. George’s Respiratory Questionnaire; VAS, visual analog scale.

#### 2.4.1 Clinical outcomes assessment

The 6-min walk distance (6-MWD), oxygen saturation, and the modified Borg scale for breathlessness of the 6-min walk test (6-MWT) were determined as the primary outcome, considering the multifaceted nature of CSBHT and HGYGT, which can affect various bodily functions beyond the respiratory system, as the 6-MWT comprehensively assesses not only respiratory function but also cardiac function, physical fitness, and psychological factors (ATS Committee on Proficiency Standards for Clinical Pulmonary Function Laboratories, 2002; Corlateanu et al., 2021). Secondary outcomes were the results of the pulmonary function test (PFT) (Graham et al., 2019), the Korean version of the Chronic Obstructive Pulmonary Disease Assessment Test (CAT) (Jones et al., 2009; Lee et al., 2010), St. George’s Respiratory Questionnaire (SGRQ) (Jones et al., 1992), modified Medical Research Council (mMRC) questionnaire (Kim et al., 2013), visual analogue scale (VAS) for dyspnea (Mador and Kufel, 1992), and the frequency of exacerbations. Details of the meaning and significance of each metric, measurement methods, interpretation of scores, and minimal clinically important difference (MCID) are described in Supplementary one in Additional file.

#### 2.4.2 Safety assessment

Adverse events were assessed according to the Common Terminology Criteria for Adverse Events version 5.0 (Freites-Martinez et al., 2021). When an adverse event occurred, the causality with the observational study was evaluated according to the World Health Organization–Uppsala Monitoring Centre criteria (Son et al., 2008). Detailed criteria for assessing safety are provided in Additional file 1.

#### 2.4.3 Syndrome differentiation assessment for COPD

The syndrome differentiation type for each patient was determined using the Syndrome Differentiation Tool for COPD, which considers seven syndrome types: wind–cold (風寒), phlegm turbidity (痰濁), lung heat (肺熱), lung deficiency (肺虛), spleen deficiency (脾虛), kidney yin deficiency (腎陰虛), and kidney yang deficiency (腎陽虛) (Lee B.-J. et al., 2012). The layered alluvial plot of syndrome differentiation change was made using the *ggalluvial* package in RStudio (v.2023.06.0, Posit team. RStudio: Integrated Development Environment for R. Posit Software, PBC, Boston, MA) (Brunson, 2020). Detailed criteria for syndrome differentiation assessment for COPD are described in Additional file 1.

#### 2.4.4 Adherence and feasibility

Dropout rates and adherence to prescribed medication regimens were recorded every month.

### 2.5 Statistical analysis

Descriptive analyses were conducted on the baseline characteristics of the study participants. Categorical variables are presented as frequency with percentage (%), and continuous variables as mean with standard deviation (mean ± SD). Values of the clinical outcome variables measured at each visit time are presented as mean ± SD and median with interquartile range. Changes in the outcome variables were measured by comparing baseline values to those at each subsequent time point. Paired *t*-tests were conducted for continuous variables if the data were normally distributed; otherwise, the Wilcoxon signed-rank test was used. To examine normality of data distribution, both a histogram and the Shapiro–Wilk test were used. Due to the exploratory nature of this study, the issue of multiple tests was not considered.

A sensitivity analysis was conducted by imputing missing values using the last observation carried forward (LOCF) method, which substitutes missing values with the last available measurement.

Safety evaluations were performed for all adverse events that occurred during the treatment period. The incidence rates of adverse events, adverse events that led to dropout, and serious adverse events were summarized by treatment group. The incidence rate of adverse events is presented for all adverse events and those related to the investigational medicines used in the observational study.

Statistical significance in all analyses was determined using a *p*-value threshold of less than 0.05. Multiple testing corrections were not applied as this was an exploratory observational study. Visualization of data distribution and changes in clinical outcomes was conducted using the *ggplot2* package in RStudio.

## 3 Results

### 3.1 Study participants and baseline characteristics

**FIGURE 1:**
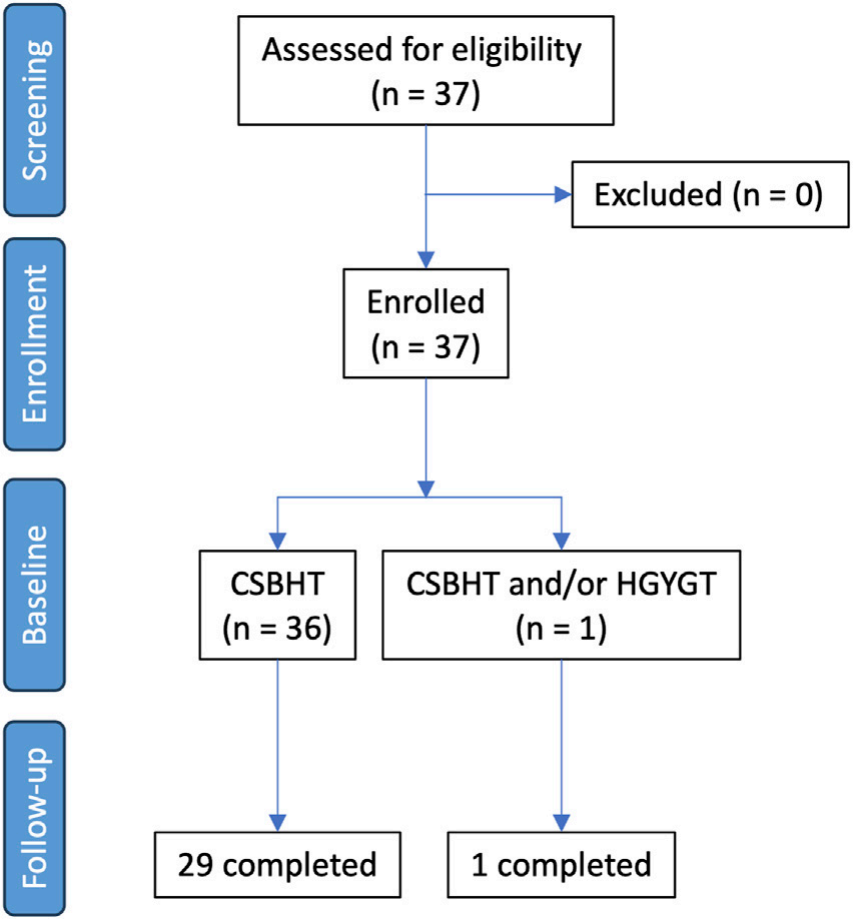
Study flow chart. CSBHT, Cheongsangboha-tang; HGYGT, Hyunggaeyeongyo-tang.

Thirty-seven patients with COPD were screened. All these patients were eligible and enrolled in this study (Figure 1). The baseline characteristics of all patients, those who were followed up for the entire treatment period, and those who dropped out are presented in Table 3. The 37 patients were included at Visit 1 (baseline). All included patients were Asian. Of these, 30 were male, with an average age of 69.5 years. The mean body mass index (BMI) was 25.0 kg/m^2^. Among the patients, 19 were never-smokers, eight were current smokers, and nine were former smokers. Additionally, 32 patients reported no alcohol consumption. The average post-bronchodilator FEV1% predicted was 56.1%, with most patients (n = 23) in GOLD stage 2, followed by 11 patients in GOLD stage 3. Twenty-four patients had an mMRC at Visit one of 0–1. Thirty-six patients had a CAT score of 10 or higher at Visit 1. All but two patients were using bronchodilator inhalers.

**TABLE 3:** Baseline characteristics of study participants.

		Complete baseline	Complete follow-up	Dropout
		(frequency (%)/mean ± SD)		
Number of patients		37	30	7
Follow-up period (days)		154.1 ± 68.4	185.2 ± 17.2	20.9 ± 33.2
Sex				
	Male	30 (81.1%)	24 (80%)	6 (85.7%)
	Female	7 (18.9%)	6 (20%)	1 (14.3%)
Age (years)				
		69.5 ± 8.0	69.9 ± 6.8	67.7 ± 12.3
	41–50	1 (2.7%)	0 (0%)	1 (14.3%)
	51–60	4 (10.8%)	3 (10%)	1 (14.3%)
	61–70	16 (43.2%)	14 (46.7%)	2 (28.6%)
	71–80	16 (43.2%)	13 (43.3%)	3 (42.9%)
BMI (kg/m^2^)				
		25.0 ± 4.2	24.6 ± 4.3	26.7 ± 3.3
	<18.5	2 (5.4%)	2 (6.7%)	0 (0%)
	18.5–22.9	8 (21.6%)	8 (26.7%)	0 (0%)
	23–24.9	12 (32.4%)	9 (30%)	3 (42.9%)
	≥25	15 (40.5%)	11 (36.7%)	4 (57.1%)
Smoking status				
	Never	19 (51%)	13 (43.3%)	6 (85.7%)
	Current	8 (22%)	8 (26.7%)	0 (0%)
	Former	9 (24%)	8 (26.7%)	1 (14.3%)
Alcohol consumption				
	No	32 (86.5%)	26 (86.7%)	6 (85.7%)
	Yes	5 (13.5%)	4 (13.3%)	1 (14.3%)
PFT				
	FEV1, L	1.6 ± 0.5	1.6 ± 0.6	1.6 ± 0.5
	FEV1, %	56.1 ± 15.9	56.9 ± 15.5	52.6 ± 18.3
	≥80 (GOLD stage 1)	1 (2.7%)	1 (3.3%)	0 (0%)
	50–79 (GOLD stage 2)	23 (67.2%)	18 (60%)	5 (71.4%)
	30–49 (GOLD stage 3)	11 (29.7%)	10 (33.3%)	1 (14.3%)
	<30 (GOLD stage 4)	2 (5.4%)	1 (3.3%)	1 (14.3%)
	FVC, L	3.0 ± 0.8	3.0 ± 0.8	2.8 ± 0.8
	FVC, %	73.2 ± 15.9	75.1 ± 14.3	65.1 ± 20.6
	FEV1/FVC, %	54.1 ± 10.9	53.5 ± 11.1	56.7 ± 10.5
mMRC				
	0–1	24 (64.9%)	18 (60%)	6 (85.7%)
	≥2	13 (35.1%)	12 (40%)	1 (14.3%)
CAT score				
	<10	1 (2.7%)	0 (0%)	1 (14.3%)
	≥10	36 (97.3%)	30 (100%)	6 (85.7%)
Comorbidities				
	Asthma	3 (8.1%)	3 (10%)	0 (0%)
	Bronchiectasis	1 (2.7%)	1 (3.3%)	0 (0%)
	Hypertension	26 (70.3%)	21 (70%)	5 (71.4%)
	Diabetes mellitus	12 (32.4%)	8 (26.7%)	4 (57.1%)
	Hyperlipidemia	15 (40.5%)	12 (40%)	3 (42.9%)
	Cerebral infarction	5 (13.5%)	3 (10%)	2 (28.6%)
	Cardiovascular diseases	15 (40.5%)	11 (36.7%)	4 (57.1%)
	Osteoarthritic diseases	8 (21.6%)	5 (16.7%)	3 (42.9%)
Medication use				
	ICS	2 (5.4%)	2 (6.7%)	0 (0%)
	SABA	2 (5.4%)	1 (3.3%)	1 (14.3%)
	LAMA	9 (24.3%)	9 (30%)	0 (0%)
	LABA + LAMA	17 (45.9%)	12 (40%)	5 (71.4%)
	ICS + LABA	8 (21.6%)	8 (26.7%)	0 (0%)
	ICS + LABA + LAMA	6 (16.2%)	5 (16.7%)	1 (14.3%)

BMI, body mass index; CAT, chronic obstructive pulmonary disease assessment test; FEV1, forced expiratory volume in the first second; FVC, forced vital capacity; GOLD, global initiative for chronic obstructive lung disease; ICS, inhaled corticosteroids; LABA, long-acting beta agonists; LAMA, long-acting muscarinic antagonists; mMRC, modified Medical Research Council; PFT, pulmonary function test; SABA, short-acting beta agonists; SD, standard deviation.

### 3.2 Follow up period and herbal preparation treatment

Of the 37 enrolled patients, 30 completed the study, while seven patients refused to participate in the study and dropped out after enrollment. Among the dropouts, two experienced mild dyspepsia and one experienced a mild headache, leading to the cessation of their participation and medication intake.

The follow-up period was 154.1 ± 68.4 days for the 37 patients overall, and more specifically, 185.2 ± 17.2 days for the 30 patients who completed the study, and 20.9 ± 33.2 days for the seven patients who dropped out. The number of patients who remained after excluding dropouts at each visit is shown in Table 4.

**TABLE 4:** Course of clinical treatment outcomes at each visit (mean ± SD).

	Visit 1 (Baseline)	Visit 2	Visit 3	Visit 4	Visit 5	Visit 6	Visit 7
	Frequency/mean ± SD						
Number of patients	37	33	31	31	30	30	30
Follow-up period (days)^a^		29.1 ± 3.4	60.7 ± 12.3	90.1 ± 15.4	122.9 ± 14.9	152.2 ± 19.7	185.2 ± 17.2
6-MWT							
Distance (m)	362.7 ± 100.5			361.6 ± 86.5			369.6 ± 68.2
Oxygen saturation (%)	94.2 ± 3.3			94.7 ± 3.1			94.9 ± 3.2
Modified Borg scale	3.3 ± 2.0			2.7 ± 1.7			2.7 ± 1.8
CAT^a^							
Total score	17.0 ± 5.0	15.9 ± 4.4	14.8 ± 3.9	15.8 ± 4.1	13.7 ± 3.5	13.7 ± 3.5	12.5 ± 3.6
Item 1 (cough)	1.6 ± 1.4	1.6 ± 1.3	1.3 ± 1.2	1.9 ± 1.3	1.7 ± 1.1	1.8 ± 1.1	1.4 ± 1.1
Item 2 (phlegm)	2.1 ± 1.3	2.2 ± 1.2	2.1 ± 1.3	2.3 ± 1.3	2.0 ± 1.1	2.0 ± 1.1	1.9 ± 1.3
Item 3 (chest tightness)	2.1 ± 0.9	1.4 ± 1.0	1.2 ± 1.0	1.1 ± 1.1	0.4 ± 0.7	0.5 ± 0.8	0.4 ± 0.7
Item 4 (exertional dyspnea)	3.8 ± 1.0	3.8 ± 1.0	4.1 ± 0.6	4.1 ± 0.8	4.0 ± 0.6	3.8 ± 0.6	3.6 ± 0.8
Item 5 (limitation of indoor activities)	1.3 ± 0.9	1.0 ± 0.7	0.9 ± 0.6	0.9 ± 0.8	0.5 ± 0.7	0.6 ± 0.8	0.4 ± 0.6
Item 6 (confidence in leaving home)	1.2 ± 0.7	1.1 ± 0.7	0.9 ± 0.7	0.8 ± 0.6	0.5 ± 0.7	0.5 ± 0.6	0.4 ± 0.6
Item 7 (quality of sleep)	2.1 ± 1.6	1.9 ± 1.4	1.7 ± 1.6	1.9 ± 1.4	1.6 ± 1.4	1.8 ± 1.4	1.7 ± 1.4
Item 8 (energy)	2.8 ± 1.0	3.0 ± 0.9	2.7 ± 0.8	2.8 ± 0.9	2.9 ± 0.8	2.7 ± 0.8	2.8 ± 0.9
SGRQ							
Total	21.2 ± 9.2	20.5 ± 6.7	20.3 ± 6.5	21.0 ± 7.3	20.8 ± 6.2	21.0 ± 6.1	20.7 ± 6.5
Symptom component	32.4 ± 10.5	32.3 ± 8.8	31.8 ± 9.5	31.6 ± 9.9	33.5 ± 12.9	30.7 ± 9.2	31.1 ± 7.9
Activity component	34.3 ± 15.6	34.2 ± 13.8	33.3 ± 12.5	34.7 ± 14.0	33.4 ± 12.2	34.9 ± 11.0	34.5 ± 12.1
Impact component	10.2 ± 6.7	9.0 ± 4.4	9.3 ± 4.7	9.8 ± 5.1	9.6 ± 4.6	10.0 ± 4.8	9.6 ± 4.8
mMRC	1.4 ± 0.9	1.3 ± 0.8	1.5 ± 0.9	1.5 ± 0.8	1.4 ± 0.8	1.5 ± 0.7	1.4 ± 0.9
VAS for dyspnea	47.5 ± 18.9	46.2 ± 19.4	45.1 ± 20.4	40.5 ± 20.1	30.7 ± 18.2	36.4 ± 20.0	28.4 ± 18.6

^a^ During the follow-up period, patients who dropped out at each visit were excluded from the analysis.

6MWT, 6-min walking test; CAT, chronic obstructive pulmonary disease assessment test; mMRC, modified Medical Research Council; SGRQ, St. George’s Respiratory Questionnaire; SD, standard deviation; VAS, visual analog scale.

CSBHT was administered to all but one patient throughout the study period. For this patient, CSBHT was given at Visits one and 2, HGYGT at Visit 3, CSBHT again at Visits 4 and 5, and both CSBHT and HGYGT at Visit 6. No prescription was provided at Visit 7. No other traditional Korean or conventional medical treatments were used during the treatment period.

### 3.3 Treatment response

**FIGURE 2:**
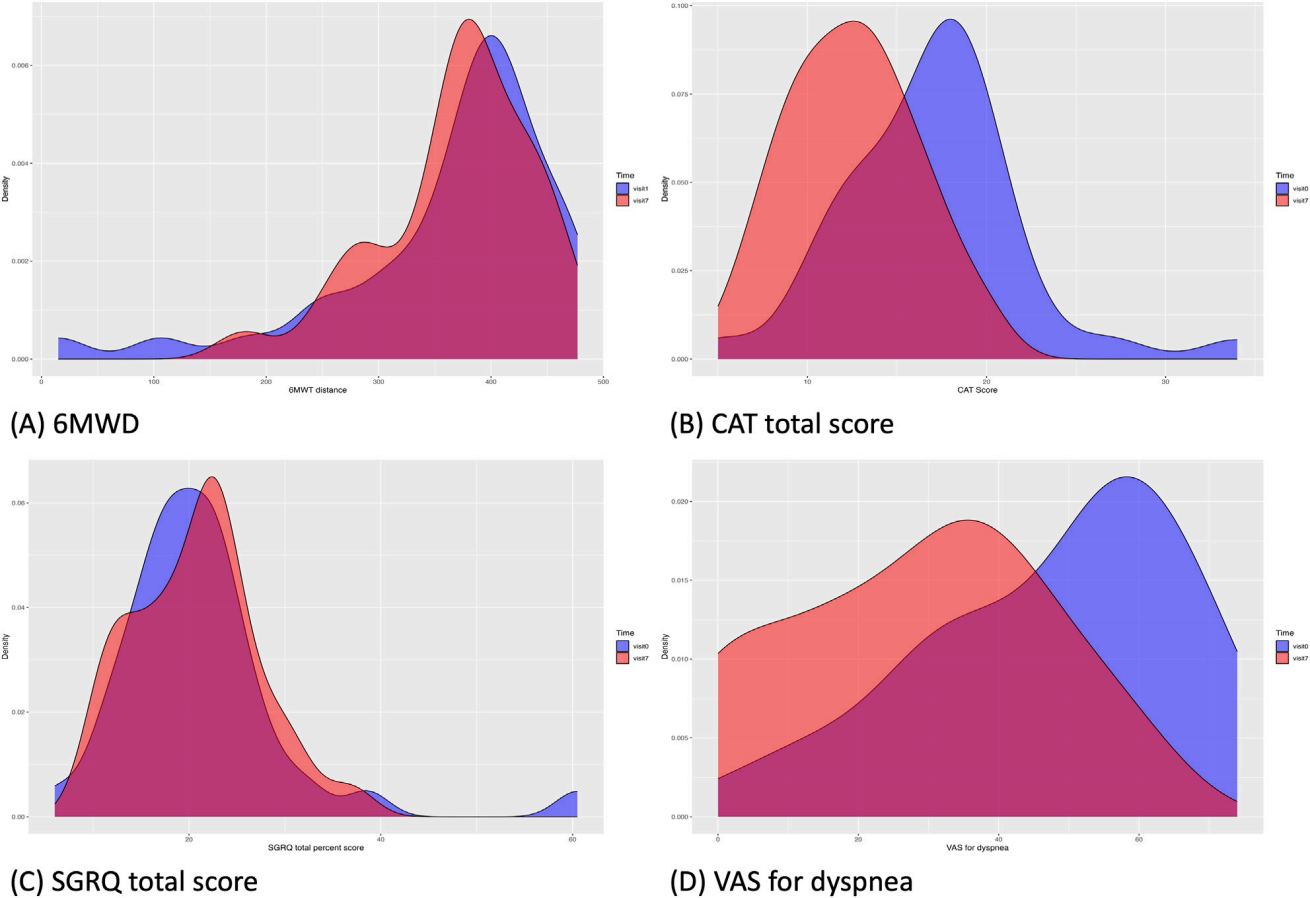
Changes in the data distribution in clinical outcomes from Visit 1 (blue) to Visit 7 (red). **(A)**, 6-MWD; **(B)**, CAT total score; **(C)**, SGRQ total score; **(D)**, VAS score for dyspnea. The density of the clinical outcome data distribution at Visit one was visualized for data of all patients included in the baseline, regardless of dropout status. 6-MWD, 6-min walking distance; CAT, chronic obstructive pulmonary disease assessment test; SGRQ, St. George’s Respiratory Questionnaire; VAS, visual analog scale.

Each assessment measure, obtained at each visit, is presented with the mean ± SD in Table 4, and the median with interquartile range in Supplementary Table S1 in Additional file 1. Table 5 presents the results of the paired *t*-test, while Supplementary Table S2 in Additional file one shows the Wilcoxon signed-rank test results. Values satisfying normality are marked with annotation symbols. The changes in data distribution density over time for each outcome measure are shown in Figure 2 for the 6-MWD, CAT score, SGRQ total score, and VAS score for dyspnea, and in Supplementary Figure S1 for every outcome measure.

**TABLE 5:** Results of the paired *t*-test for changes in clinical outcomes at each visit time, compared to Visit 1 (baseline).

	Visit 2	Visit 3	Visit 4	Visit 5	Visit 6	Visit 7
	Mean difference (95% confidence interval)					
6-MWT						
Distance			−3.0 (−34.7, 28.8)			6.7 (−17.9, 31.2)
Oxygen saturation			0.6 (−0.4, 1.6)^a^			0.8 (0.0, 1.7)^b^ ^,^ ^a^
Modified Borg scale			−0.3 (−1.0, 0.5)^a^			−0.3 (−1.0, 0.4)
CAT						
Total score	−0.3 (−1.5, 0.8)^a^	−1.5 (−2.8, −0.2)^b^ ^,^ ^a^	−0.5 (−2.1, 1.1)^a^	−3.0 (−4.5, −1.5)^b^ ^,^ ^a^	−3.0 (−4.6, −1.3)^b^	−4.1 (−5.5, −2.7)^b^ ^,^ ^a^
Item1	0.2 (−0.2, 0.6)	−0.2 (−0.6, 0.3)^a^	0.4 (−0.1, 1.0)	0.2 (−0.3, 0.7)^a^	0.3 (−0.2, 0.9)	−0.1 (−0.7, 0.4)
Item2	0.2 (−0.3, 0.6)	0.0 (−0.4, 0.4)	0.2 (−0.3, 0.7)	−0.1 (−0.5, 0.4)	−0.1 (−0.5, 0.3)	−0.2 (−0.7, 0.3)
Item3	−0.5 (−0.8, −0.3)^b^	−0.8 (−1.1, −0.4)^b^	−0.8 (−1.1, −0.5)^b^	−1.6 (−1.9, −1.2)^b^	−1.5 (−1.9, −1.1)^b^	−1.6 (−2.0, −1.2)^b^
Item4	0.1 (−0.3, 0.4)	0.4 (0.0, 0.7)^b^	0.4 (0.1, 0.7)^b^	0.2 (−0.2, 0.5)	0.0 (−0.3, 0.4)	−0.2 (−0.5, 0.2)
Item5	−0.2 (−0.5, 0.1)	−0.2 (−0.5, 0.0)	−0.3 (−0.6, 0.0)	−0.7 (−1.0, −0.4)^b^	−0.6 (−0.9, −0.3)^b^	−0.8 (−1.1, −0.5)^b^
Item6	−0.1 (−0.4, 0.2)	−0.3 (−0.5, 0.0)	−0.4 (−0.6, −0.1)^b^	−0.6 (−0.9, −0.4)^b^	−0.7 (−1.0, −0.4)^b^	−0.8 (−1.0, −0.5)^b^
Item7	−0.1 (−0.4, 0.3)	−0.3 (−0.7, 0.1)	−0.1 (−0.6, 0.4)	−0.4 (−0.9, 0.1)	−0.2 (−0.8, 0.4)^a^	−0.3 (−0.8, 0.1)
Item8	0.2 (−0.2, 0.5)	−0.1 (−0.5, 0.2)	0.0 (−0.4, 0.4)	0.0 (−0.3, 0.4)	−0.2 (−0.5, 0.2)	−0.1 (−0.4, 0.2)
SGRQ						
Total	0.5 (−0.5, 1.5)	0.3 (−0.5, 1.1)	0.9 (−0.7, 2.6)	0.3 (−0.7, 1.2)	0.5 (−0.5, 1.5)	0.2 (−0.9, 1.3)
Symptom component	1.5 (−0.1, 3.1)	0.8 (−0.9, 2.5)^a^	0.6 (−1.3, 2.4)^a^	1.9 (−2.6, 6.5)	−0.9 (−2.8, 1.1)^a^	−0.5 (−3.1, 2.1)^a^
Activity component	1.1 (−1.2, 3.5)	0.2 (−1.9, 2.3)	1.6 (−1.8, 5.0)	−0.6 (−2.9, 1.6)	0.8 (−1.0, 2.7)	0.4 (−1.5, 2.3)
Impact component	−0.2 (−0.8, 0.3)	0.2 (−0.4, 0.7)	0.6 (−0.6, 1.8)	0.3 (−0.8, 1.4)	0.6 (−0.3, 1.6)	0.3 (−0.8, 1.3)
mMRC	−0.1 (−0.2, 0.1)	0.1 (−0.1, 0.4)	0.1 (−0.1, 0.3)	0.0 (−0.3, 0.3)	0.1 (−0.3, 0.4)	0.0 (−0.3, 0.4)
VAS for dyspnea	−1.1 (−7.4, 5.3)^a^	−1.3 (−8.8, 6.2)	−5.9 (−12.8, 1.0)^a^	−17.2 (−23.9, −10.4)^b^ ^,^ ^a^	−11.5 (−19.4, −3.5)^a^	−19.5 (−26.3, −12.7)^a^

^a^ , statistically significant (*p* < 0.05).

^b^ , Satisfaction of normality in the data distribution.

6MWD, 6-min walking test; CAT, chronic obstructive pulmonary disease assessment test; SGRQ, St. George’s Respiratory Questionnaire; mMRC, modified Medical Research Council; VAS, visual analog scale.

#### 3.3.1 Primary outcome

From Visit one to Visit 7, the 6-MWD, the primary outcome of the study, changed from 362.7 ± 100.5 m to 369.6 ± 68.2 m, with no statistically significant difference observed.

#### 3.3.2 Secondary outcomes

##### 3.3.2.1 PFT

All patients underwent PFT at Visit 1. Due to the coronavirus disease 2019 pandemic, many patients were reluctant to undergo the planned PFTs at Visits 4 and 7; only 4 patients completed the PFT at Visit 4 and 14 patients at Visit 7. Only one patient completed all the planned PFTs.

##### 3.3.2.2 Oxygen saturation and modified borg scale after 6-MWT

Oxygen saturation changed from 94.2% ± 3.3% to 94.9% ± 3.2%, and the modified Borg scale from 3.3 ± 2.0 to 2.7 ± 1.8. No statistically significant differences were observed for any of the items.

##### 3.3.2.3 CAT score

**FIGURE 3:**
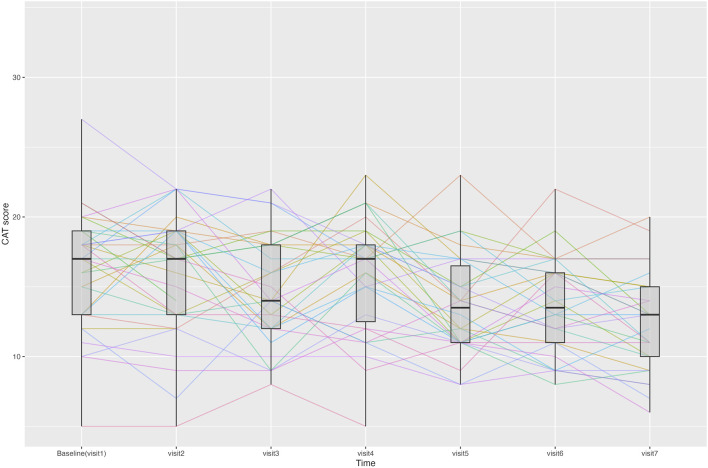
Line graph showing changes in Chronic Obstructive Pulmonary Disease Assessment Test (CAT) scores for each patient at each time point. Median and interquartile range of the CAT scores are displayed using boxplots.

Changes in the CAT scores are presented in Figure 3. From Visit one to Visit 7, the CAT total score changed from 17.0 ± 5.0 to 12.5 ± 3.6. The changes in scores from Visit 1, excluding Visit 6, satisfied a normal distribution. The average change was −4.1 (95% CI, −5.5 to −2.7) at Visit 7, with statistical significance in the change observed continuously after Visit 5. Twenty-four patients demonstrated a decrease of >2 points in their CAT scores from Visit one to Visit 7, thus achieving the MCID (Kon et al., 2014).

During the treatment period, changes in the individual CAT items did not satisfy normality assumptions. Results from the Wilcoxon signed-rank test indicated that the changes in individual items of the CAT, specifically items 3 (chest tightness), 5 (limitation of indoor activities), and 6 (confidence in leaving home), were statistically significant after treatment. Statistical significance in the change of these items was consistently achieved, starting at Visit 2 for item 3, Visit 5 for item 5, and Visit 4 for item 6.

##### 3.3.2.4 SGRQ

From Visit one to Visit 7, the SGRQ total score changed from 21.2 ± 9.2 to 20.7 ± 6.5, the symptom component from 32.4 ± 10.5 to 31.1 ± 7.9, and the activity component from 34.3 ± 15.6 to 34.5 ± 12.1, with no statistically significant changes observed.

##### 3.3.2.5 mMRC

From Visit one to Visit 7, the mMRC scale remained unchanged at 1.4 ± 0.9, with no statistically significant changes observed.

##### 3.3.2.6 VAS for dyspnea

**FIGURE 4:**
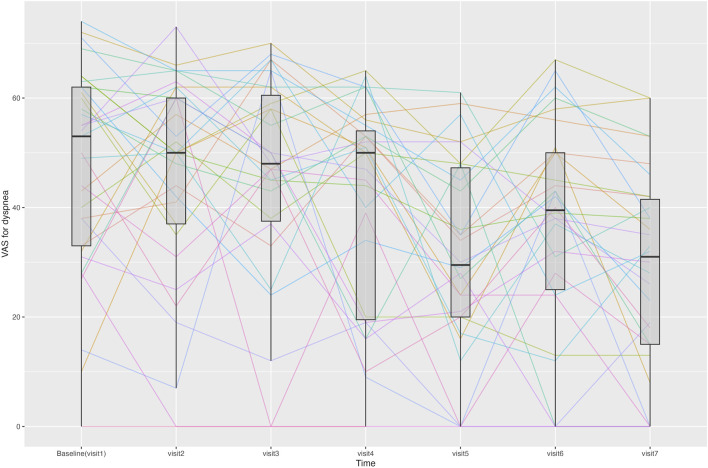
Line graph showing changes in the visual analogue scale (VAS) score for dyspnea for each patient at each time point. Median and interquartile range of the VAS scores for dyspnea are displayed using boxplots.

The change in the VAS score for dyspnea from Visit one to Visit 7 is shown in Figure 4. During the treatment period, the changes in the VAS score for dyspnea from Visit one to each subsequent visit met the criteria for normal distribution, except for Visit 3. From Visit one to Visit 7, the VAS score for dyspnea changed significantly from 47.5 ± 18.9 to 28.4 ± 18.6. Changes were statistically significant from Visit 5. Twenty-three patients experienced a decrease of more than 10 points in their VAS score for dyspnea from Visit one to Visit 7, thus achieving an MCID (Ries, 2005).

##### 3.3.2.7 Sensitivity analysis of CAT score and VAS for dyspnea with LOCF method

After imputing missing values using the LOCF method, changes over time in the CAT and VAS scores for dyspnea are presented in Table 6. Compared to Visit 1, at visit 7, the CAT score decreased by an average of 3.5 points, and the VAS score for dyspnea decreased by an average of 16.5 points, both of which remained statistically significant. The changes in both CAT and VAS scores for dyspnea were consistently statistically significant after Visit 5.

**TABLE 6:** Results of the paired *t*-tests for changes in CAT score and VAS score for dyspnea at each visit time, compared to Visit 1, utilizing the Last Observation Carried Forward imputation method to address missing data.

	Visit 2	Visit 3	Visit 4	Visit 5	Visit 6	Visit 7
Mean difference (95% confidence interval)						
CAT total score	−0.3 (−1.3, 0.7)	−1.4 (−2.5, −0.3)^a^	−0.5 (−1.9, 0.8)	−2.6 (−3.8, −1.3)^a^	−2.5 (−3.9, −1.2)^a^	−3.5 (−4.7, −2.2)^a^
VAS for dyspnea	−0.9 (−6.6, 4.7)	−1.8 (−8.1, 4.6)	−5.6 (−11.5, 0.2)	−14.6 (−20.5, −8.8)^a^	−10.0 (−16.6, −3.4)^a^	−16.5 (−22.5, −10.6)^a^

^a^ , statistically significant (*p* < 0.05).

CAT, chronic obstructive pulmonary disease assessment test; VAS, visual analogue scale.

##### 3.3.2.8 Frequency of COPD exacerbation

None of the patients experienced exacerbations during the treatment period.

##### 3.3.2.9 Syndrome differentiation assessment

**FIGURE 5:**
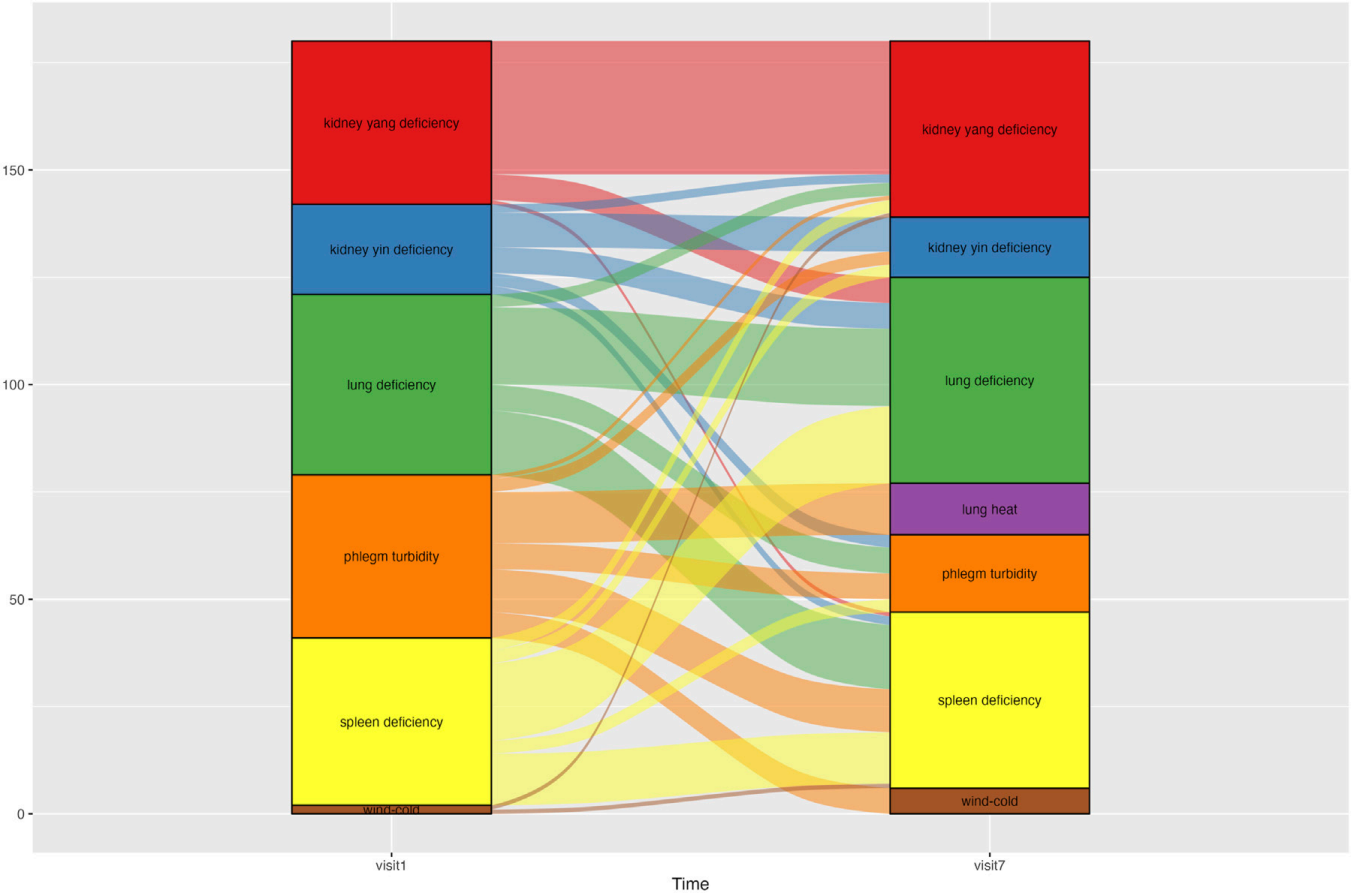
Alluvial graph showing changes in patients’ syndrome differentiation type from Visit one to Visit 7. An alluvial graph was drawn with exclusion of the patients who dropped out of the study. Patients were categorized into one of three syndrome differentiation types at each time point. The graph column for Visit one consisted of 45 syndrome differentiation data points from 30 patients. The column for Visit 7 comprised 39 syndrome differentiation data points from 30 patients.

Changes in syndrome differentiation during the treatment period are shown in Figure 5. Each patient was classified into one to three of the seven possible syndrome types. At Visit 1, 12 patients were classified as having spleen deficiency, 11 as having lung deficiency, and nine as having phlegm turbidity. At Visit 7, 10 patients were classified as having spleen deficiency, nine had lung deficiency and kidney yang deficiency. From Visits one to 7, the number of patients classified as having phlegm turbidity showed the most significant decrease, from nine to four.

##### 3.3.2.10 Safety assessment

During the course of the study, among the patients administered CSBHT, two instances of grade 1 dyspepsia, and one instance of grade 1 headache occurred, leading all three patients to discontinue their medication and study participation (Supplementary Table S3 in Additional file 1). The symptoms resolved after discontinuation of medication. No serious adverse events related to the medications used in this study were observed. No significant changes were observed in liver and kidney function or in hematological test results during the treatment period.

## 4 Discussion

### 4.1 Summary of findings

We observed the treatment response to CSBHT in patients with COPD over an average of 6 months in a prospective, proof-of-concept study. Thirty-seven patients were included at baseline, of whom 30 completed the study. CSBHT was administered to all patients except one, who received either CSBHT, HGYGT, or both at different visits. At Visit 7, significant improvements were observed, with the CAT score decreasing from an average of 17.0 to 12.5, and the VAS score for dyspnea decreasing from 47.5 to 28.4. Even after imputing missing values due to patient drop-out, using the LOCF method, the mean difference decreased slightly, yet statistical significance was consistently observed from Visit 5 onwards. No significant changes were observed in the 6-MWT, SGRQ, or mMRC scores. No exacerbations or serious adverse events occurred during treatment. Additionally, changes in syndrome differentiation were noted before and after treatment.

### 4.2 Therapeutic potential of CSBHT for respiratory diseases, including COPD

CSBHT has primarily been used for respiratory diseases, such as chronic cough and asthma, particularly when accompanied by chronic inflammatory phlegm, in clinical practice in East Asian traditional medicine (EATM) (Baek et al., 2016b). The formulation of CSBHT is focused on phlegm-dispelling (祛痰) and moistening the airways (滋陰潤肺), making it particularly suitable for treating various chronic respiratory diseases, especially those assessed as deficiency patterns (虛證) (Kang et al., 2022). Studies have been conducted on modified CSBHT, which have demonstrated its ability to attenuate the accumulation of immune cells in the bronchoalveolar lavage fluid (BALF) significantly in a mouse model of lipopolysaccharide-induced lung neutrophilia (Lee H. et al., 2012). Additionally, modified CSBHT inhibited immune cell infiltration into the airways and decreased interleukin-6, tumor necrosis factor-***α***, and monocyte chemoattractant protein-1 levels in BALF in a mouse model of cigarette smoke-induced lung inflammation (Jung et al., 2013). The antitussive, expectorant, anti-inflammatory, antioxidant, and anti-asthmatic effects of the individual botanical drugs in CSBHT on respiratory diseases have been also explored (Lee et al., 2005; 2019; Quan et al., 2021; Wei et al., 2023).

### 4.3 Dyspnea as a potential therapeutic target of CSBHT in patients with COPD

In this study, significant improvements were observed in the CAT and VAS scores for dyspnea in patients with COPD, over an average treatment period of 6 months, whereas no significant changes were noted in the 6-MWT, mMRC, and SGRQ scores. In an observational study conducted in South Korea, Lyu et al. administered adjuvant traditional Korean medical therapy, including Palmijihwang-tang (PMJHT) and acupuncture, alongside conventional medicine, to ten patients with COPD. After 12 weeks, significant improvements were reported, with the mMRC decreasing from an average of 1.60 to 0.89, the total SGRQ score decreasing from 42.87 to 35.25, and the SGRQ impact component decreasing from 24.67 to 16.51, while no significant difference was observed in the CAT score (Lyu et al., 2022).

In this study, CSBHT has been shown to affect certain aspects of dyspnea in patients with COPD, although no significant improvements have been observed in exertional dyspnea, indicating that CSBHT holds particular therapeutic potential in COPD treatment (Sigurgeirsdottir et al., 2019). Dyspnea is the most commonly experienced subjective symptom among COPD patients and is associated with anxiety and fear of breathlessness, significantly affecting patients’ quality of life. Dyspnea has been identified to possess four different somatic descriptors of breathlessness: 1) a perceived sense of increased work of effort; 2) a sense of chest tightness; 3) air hunger or an uncomfortable urge to breathe; and 4) unsatisfied inspiration (O’Donnell et al., 2007). However, standardized dyspnea assessment tools evaluate different aspects of dyspnea, which highlights that appropriate assessment tools should be used, depending on the therapeutic and research objectives (Meek, 2004; Ong, 2021). Contrary to the findings of Lyu et al., this study observed no significant changes in the mMRC and modified Borg scale; nevertheless, significant improvements were found in the VAS score for dyspnea and the CAT score, particularly for the item assessing chest tightness. This may be because, compared with the participants in the study by Lyu et al., this study had a high proportion of patients with accompanying cardiovascular disease, which might additionally influence dyspnea. Furthermore, CSBHT used in this study may have affected specific aspects of dyspnea through mechanisms different from those of PMJHT. Further studies are needed to derive and apply optimal herbal formulations according to the disease state and the therapeutic targets in COPD.

### 4.4 Sufficient treatment duration of herbal preparation for patients with COPD

Significant improvements in the CAT score and VAS for dyspnea were observed, starting from Visit 5 (mean follow-up period, 122.9 days) in this study. Previous studies on the effects of herbal preparations on COPD have shown that these effects tend to vary with the duration of administration. In a systematic review by Kwon et al., who compared herbal preparation for COPD to a placebo, a subgroup analysis based on treatment duration found that even administration lasting less than 2 weeks could improve pulmonary function and 6-MWD in cases of acute exacerbation of COPD. Among the ten studies of stable and unclear COPD, all but one study with treatment durations less than 2 months showed no significant improvement. In contrast, two studies with treatment periods exceeding 3 months reported significant improvements. An observational study by Lyu et al. also found no significant changes at 4 and 8 weeks in 10 patients with COPD treated with PMJHT; however, significant improvements in the mMRC and SGRQ scores were observed at 12 weeks. Additionally, a retrospective study by Baek et al., which analyzed the effects of CSBHT in patients with chronic respiratory diseases, including COPD, found a significant decrease in eosinophil levels in patients treated for more than 3 months. These results collectively suggested that, while acute exacerbations may improve with relatively short periods of herbal preparation treatment, stable COPD may require a treatment duration of at least 3 months. This finding was consistent with the results of this study.

### 4.5 Optimal treatment strategy considering changes in syndrome differentiation of COPD during CSBHT treatment

In this study, after excluding patients who had dropped out, the predominant syndrome types at baseline were splenic deficiency, pulmonary deficiency, and phlegm turbidity. However, during the CSBHT treatment period, some patients experienced changes in their syndrome differentiation, such that spleen deficiency, pulmonary deficiency, and kidney yang deficiency accounted for the majority of syndromes by Visit 7. Syndrome differentiation is a method used in EATM that seeks to stratify patients with COPD further according to EATM-specific procedures, by acquiring, analyzing, and integrating clinical information to determine the therapeutic strategy and corresponding modality of interventions, in order to optimize treatment efficacy (Jiang et al., 2012). EATM’s syndrome differentiation not only focuses on organs directly associated with a specific disease but also considers interrelationships among other organs in diagnosis and treatment. This approach aligns with EATM’s multifaceted perspective on disease. This study observed a decrease in the syndrome type of phlegm turbidity and an increase in the number of patients with kidney yang deficiency after CSBHT, which indicates changes in patient syndrome types. Such changes in syndrome differentiation serve as the basis for modifying primary therapeutic goals and prescriptions. The unique approach of EATM, which uses different therapeutic goals, strategies, and prescriptions based on the varying symptoms and signs following specific patterns in each patient, allows for the development of flexible treatment strategies. This approach not only offers an alternative for non-responders to standard treatments or for whom standard treatment is challenging due to multi-comorbidity, but can also be actively used to prevent COPD progression from precursor conditions (Han et al., 2021). To achieve this, extensive research into the distribution of syndrome types across various stages and pathophysiological changes before and after COPD diagnosis is needed, to allow identification of the optimal herbal formulation that exhibits the maximum effect for each stage and pathology.

### 4.6 Strengths and limitations

This study has several strengths. It prospectively observed patients over a relatively long period of 6 months of CSBHT administration. Although previous prospective observational studies have evaluated the effects of herbal preparation on COPD, they were conducted over a 12-week period, whereas this study extended the observation to 6 months, suggesting the need for further research on the optimal duration of herbal preparation administration for cases of stable COPD. Additionally, we utilized quantitative tools, such as the 6-MWT, mMRC score, CAT score, VAS score for dyspnea, and SGRQ score, to evaluate the effects of CSBHT on COPD comprehensively, and employed a Syndrome Differentiation Questionnaire to assess and monitor changes in patients’ subtypes quantitatively from the perspective of traditional medicine.

However, this study had several limitations. Being an observational study without a control group, it is challenging to differentiate whether the observed effects are due to the administered medication, external factors, or coincidence, and the relatively small number of included patients increases the potential for bias. Hence, an RCT is warranted to evaluate the effects of CSBHT on COPD. Additionally, in this study, a large number of patients had indications for CSBHT, allowing for a focused examination of the response of COPD patients to CSBHT. However, research on HGYGT was not feasible based on our cohort, given that HGYGT is primarily prescribed to younger patients with prominent inflammatory symptoms, while almost none of the patients presenting at our clinic met these criteria. This situation was worsened during the COVID-19 pandemic. Furthermore, PFT measurements were not conducted as planned, due to the COVID-19 pandemic; consequently, further research is needed to address this gap. Furthermore, while we used the mMRC scale for dyspnea assessment, future studies should consider the Baseline Dyspnea Index for its multidimensional evaluation or the Transitional Dyspnea Index for its higher sensitivity to changes.

## 5 Conclusion

In this study, CSBHT was administered to patients with COPD for approximately 6 months, and significant improvements in the CAT score and VAS score for dyspnea were observed from around the 4-month time-point, suggesting the potential effects of CSBHT on certain aspects of dyspnea in patients with COPD. Additionally, we confirmed that this herbal preparation treatment was relatively safe, as no significant adverse events were observed during therapy. However, because changes in residual symptoms and syndrome differentiation were observed during the course of treatment, adjustments to the therapeutic prescription may be required as the treatment progresses, indicating the need for further research. Our findings also highlight the need to apply appropriate outcome measures with optimal herbal formulations and treatment durations when assessing the efficacy of herbal preparations for COPD; this should be considered in subsequent research.

## Data Availability

The raw data supporting the conclusions of this article will be made available by the authors, without undue reservation.

## Abbreviations

6-MWT, 6-min walking test; BALF, bronchoalveolar lavage fluid; CAT, Chronic Obstructive Pulmonary Disease Assessment Test; COPD, chronic obstructive pulmonary disease; CSBHT, Cheongsangboha-tang (Qing Shang Bu Xia Tang); EATM, East Asian traditional medicine; GOLD, Global Initiative for Chronic Obstructive Lung Disease; HGYGT, Hyunggaeyeongyo-tang (Jing Jie Lian Qiao Tang); LOCF, last observation carried forward; mMRC, modified Medical Research Council; PFT, pulmonary function test; PMJHT, palmijihwang-tang (PMJHT); RCT, randomized controlled trial; SGRQ, St. George’s Respiratory Questionnaire.
