# Supplementary material for: Prospective proof-of-concept observational RESEarch about traditional herbal preparation Treatment for Chronic Obstructive Pulmonary Disease (RESET-COPD-1)
Source: Front Pharmacol. 2024 Sep 26;15:1437253. doi: 10.3389/fphar.2024.1437253 (PMC11464318; doi:10.3389/fphar.2024.1437253)
Supplement: Supplementary file 1 [file DataSheet1.docx]

**Supplementary 1. Details of clinical outcome and safety assessment**

**1) Primary outcome**

***6-Minute Walk Test (6MWT)***

The 6MWT was administered every three months. The 6MWT measures the distance a patient can walk quickly on a flat, hard surface in a period of six minutes (6-Minute Walk Distance, 6MWD). Oxygen saturation was measured using a pulse oximeter, and breathless was assessed using the modified Borg scale after a 6-minute walk. 6MWT assesses the overall and integrated response of all systems involved during exercise, including the respiratory, cardiovascular, systemic and peripheral circulation, hematologic, neuromuscular, and muscle metabolism [1]. The minimal clinically important difference (MCID) for improvement in the 6MWT for patients with severe COPD was determined to be 26±2 meters [2].

**2) Secondary outcomes**

***Pulmonary function test (PFT)***

Pulmonary function tests were performed every three months. Spirometry is the most objective and reproducible method for assessing airflow limitation. Through spirometry, Forced Vital Capacity (FVC) and Forced Expiratory Volume in one second (FEV_1_) are measured, and the FEV_1_/FVC ratio is calculated. For the FEV_1_/FVC ratio, the lower the value, the more severe the airflow limitation, with values below 0.70 defining the presence of airflow limitation [3]. The MCID for improvement in trough FEV_1_ has been studied to be 100ml [4].

***COPD Assessment Test (CAT)***

CAT score was taken every month. The CAT is a tool used to evaluate the quality of life in patients with COPD. CAT comprises items 1 through 8, each scored from 0 to 5, evaluating the following components in order: 1) cough, 2) phlegm, 3) chest tightness, 4) exertional dyspnea, 5) limitation of indoor activities, 6) confidence leaving home, 7) quality of sleep, and 8) energy. The sum of all item scores constitutes the CAT score. The CAT is a concise version of the widely used Saint George's Respiratory Questionnaire (SGRQ), and it has been validated to correlate with the SGRQ and demonstrate good internal consistency [5]. Higher CAT scores indicate poorer quality of life, with the worst state being 40 points and the best state being 0 points. The estimated MCID for improvement in the CAT score has been determined to be 2 points [6]. In this study, the Korean version of the CAT was utilized [7].

***St. George’s Respiratory Questionnaire (SGRQ)***

The SGRQ was measured every month. It is a validated tool for assessing the quality of life in patients with COPD, with proven validity and reliability, including a version validated for use in Korean. It consists of three domains: symptoms, activity, and impacts, comprising a total of 50 items. The SGRQ is scored from 0 to 100, with lower scores indicating a higher quality of life [8]. The MCID for improvement in the SGRQ has been determined to be 4 units [9].

***Modified Medical Research Council (mMRC) Questionnaire***

The mMRC Questionnaire was used to assess the severity of breathlessness every month. The mMRC scores range from 0 to 4, with higher scores indicating more severe dyspnea. An mMRC score of ≥2 is considered the cut-point for separating "less breathlessness" from “more breathlessness” [10]. The dyspnea scores obtained using the mMRC Questionnaire have been found to correlate well with other assessment methods evaluating the patient's health status. Higher scores of mMRC Questionnaire are associated with a worse prognosis and increased risk of mortality [11].

***Visual Analog Scale (VAS) for dyspnea***

The VAS was utilized as a tool to measure the severity of breathlessness, and it was assessed every month [12]. The MCID for improvement in the VAS for dyspnea was determined to be 10 to 20 units through a distribution-based method [13].

***The frequency of COPD exacerbations***

The frequency with severity of COPD exacerbations were assessed and recorded every month.

**3) Syndrome Differentiation Assessment for COPD**

The Syndrome Differentiation Tool for COPD, developed as an indicator for distinguishing the pattern of COPD patients and assessing the clinical response to the treatment, is categorized into seven types of syndrome: wind-cold (風寒), phlegm turbidity (淡濁), lung heat (肺熱), lung deficiency (肺虛), spleen deficiency (脾虛), kidney yin deficiency (腎陰虛), and kidney yang deficiency (腎陽虛). The score of each syndrome was measured with nine items on a 5-point Likert scale [14]. The primary syndrome for each patient was determined by applying a weighted formula to derive scores, with the highest-scoring syndrome identified as the primary one. The layered alluvial plot of syndrome differentiation change was made using the *ggalluvial* package with RStudio (v.2023.06.0, Posit team. RStudio: Integrated Development Environment for R, Posit Software, PBC, Boston, MA) [15].

**4) Safety assessment**

***Adverse event***

Adverse events were assessed and reported every month according to the Common Terminology Criteria for Adverse Events version 5.0 (CTCAE v5.0), grading them as Grade 1 (Mild), Grade 2 (Moderate), Grade 3 (Severe or medically significant), Grade 4 (Life-threatening consequences), and Grade 5 (Death related to AE) [16]. When an adverse event occurred, the causality with the observational study was evaluated according to the World Health Organization-Uppsala Monitoring Centre (WHO-UMC) criteria, which include: Certain, Probable/Likely, Possible, Unlikely, Conditional/Unclassified, and Unassessable/Unclassifiable, with the researcher's opinion being documented [17].

Table S 1 Clinical outcome changes with median and interquartile range.

|  | **Visit 1 (Baseline)** | **Visit 2** | **Visit 3** | **Visit 4** | **Visit 5** | **Visit 6** | **Visit 7** |
| --- | --- | --- | --- | --- | --- | --- | --- |
|  | Frequency / mean±*SD* / median [interquartile range] | | | | | | |
| **Number of patients** | 37 | 33 | 31 | 31 | 30 | 30 | 30 |
| **Follow-up period** |  | 29.1±3.4 | 60.7±12.3 | 90.1±15.4 | 122.9±14.9 | 152.2±19.7 | 185.2±17.2 |
| **6MWT** |  |  |  |  |  |  |  |
| **Distance (m)** | 392 [343, 420] |  |  | 384 [305.5, 422] |  |  | 379 [343.8, 415. 3] |
| **Oxygen saturation (%)** | 95 [92, 97] |  |  | 96 [94, 97] |  |  | 96 [93.3, 97] |
| **Modified Borg scale** | 3 [2, 4] |  |  | 3 [2, 4] |  |  | 3 [2, 3. 8] |
| **CAT** |  |  |  |  |  |  |  |
| **Total score** | 17 [ 13, 19] | 17 [13, 19] | 14 [12, 18] | 17 [12.5, 18] | 13.5 [11, 16.5] | 13.5 [11, 16] | 13 [10, 15] |
| **Item 1** | 1 [0, 3] | 1 [1, 3] | 1 [0, 2] | 2 [0.5, 3] | 2 [1, 2.8] | 2 [1, 3] | 1 [0.3, 2] |
| **Item 2** | 2 [1, 3] | 2 [1, 3] | 2 [1, 3] | 3 [1.5, 3] | 2 [1, 3] | 2 [1, 3] | 2 [1, 3] |
| **Item 3** | 2 [2, 3] | 1 [1, 2] | 1 [0, 2] | 1 [0, 2] | 0 [0, 1] | 0 [0, 1] | 0 [0, 1] |
| **Item 4** | 4 [4, 4] | 4 [4, 4] | 4 [4, 4] | 4 [4, 4.5] | 4 [4, 4] | 4 [3.3, 4] | 4 [3, 4] |
| **Item 5** | 1 [1, 2] | 1 [1, 1] | 1 [1, 1] | 1 [0, 1] | 0 [0, 1] | 0.5 [0, 1] | 0 [0, 1] |
| **Item 6** | 1 [1, 2] | 1 [1, 1] | 1 [0, 1] | 1 [0, 1] | 0 [0, 1] | 0 [0, 1] | 0 [0, 1] |
| **Item 7** | 1 [1, 4] | 1 [1, 3] | 1 [1, 3] | 1 [1, 3] | 1 [1, 3] | 1 [1, 3] | 1 [0.25, 3] |
| **Item 8** | 3 [2, 4] | 3 [3, 4] | 3 [2, 3] | 3 [2, 4] | 3 [3, 3] | 3 [2, 3] | 3 [2, 3] |
| **SGRQ** |  |  |  |  |  |  |  |
| **Total** | 19.6 [16.6, 23.4] | 20.3 [16.8, 24] | 19.9 [16.3, 24] | 20.7 [16.8, 25.9] | 20.1 [17.075, 25.1] | 20.55 [17.0, 25.7] | 21.6 [16.8, 23.8] |
| **Symptom component** | 32.1 [27.1, 38.5] | 32.1 [28, 38.5] | 32.6 [26.7, 36.6] | 32.2 [26.1, 37.8] | 34.35 [28. 3, 38.4] | 32.15 [26, 36.5] | 32.2 [26.7, 36.3] |
| **Activity component** | 29.6 [29.6, 41.8] | 35.8 [29.6, 41.8] | 35.8 [29.6, 41.8] | 35.8 [29.6, 41.8] | 35.8 [29. 6, 41.8] | 35.8 [29.6, 41.8] | 35.8 [29.6, 41.8] |
| **Impact component** | 9.3 [6.2, 13.3] | 6.2 [6.2, 13.3] | 6.2 [6.2, 13.3] | 6.2 [6.2, 13.3] | 7.75 [6.2, 13.3] | 6.2 [6.2, 13. 5] | 6.2 [6.2, 13.3] |
| **mMRC** | 1 [1, 2] | 1 [1, 2] | 1 [1, 2] | 2 [1, 2] | 1 [1, 2] | 1 [1, 2] | 1 [1, 2] |
| **VAS for dyspnea** | 53 [33, 62] | 50 [37, 60] | 48 [37.5, 60. 5] | 50 [19.5, 54] | 29.5 [20, 47. 3] | 39.5 [25, 50] | 31 [15, 41. 5] |

Table S 1

SD, standard deviation; 6MWT, 6 minutes walking test; CAT, chronic obstructive pulmonary disease assessment test; SGRQ, St. George’s respiratory questionnaire; mMRC, modified Medical Research Council; VAS, visual analogue scale.

Table S 2 Results of the Wilcoxon signed rank test for changes in clinical outcomes at each visit time compared to Visit 1.

|  | **Visit 2** | | **Visit 3** | | **Visit 4** | | **Visit 5** | | **Visit 6** | | **Visit 7** | |
| --- | --- | --- | --- | --- | --- | --- | --- | --- | --- | --- | --- | --- |
|  | W statistic (*P*-value) | | | | | | | | | | | |
| **6MWT** |  |  |  |  |  |  |  |  |  |  |  |  |
| **Distance** |  |  |  |  | 207.5 (0.61) | |  |  |  |  | 210.5 (0.66) | |
| **Oxygen saturation** |  |  |  |  | 107.5 (0.23)† | |  |  |  |  | 103.5 (0.07)† | |
| **Modified Borg scale** |  |  |  |  | 147 (0.51)† | |  |  |  |  | 115.5 (0.41) | |
| **CAT** |  |  |  |  |  |  |  |  |  |  |  |  |
| **Total score** | 218 (0.49)† | | 354 (0.04)† | | 233 (0.74)† | | 356.5 (<0.001)† | | 379 (<0.001) | | 409.5 (<0.0001)† | |
| **Item 1** | 97 (0.33) | | 147.5 (0.49)† | | 76 (0.17) | | 115.5 (0.49)† | | 96 (0.31) | | 96.5 (0.64) | |
| **Item 2** | 111 (0.4) | | 155 (0.89) | | 91.5 (0.38) | | 91 (0.82) | | 122 (0.51) | | 125 (0.44) | |
| **Item 3** | 140 (<0.01) | | 178 (<0.001) | | 183.5 (<0.001) | | 351 (<0.00001) | | 345.5 (<0.0001) | | 363 (<0.0001) | |
| **Item 4** | 78 (0.75) | | 66.5 (0.07) | | 10 (0.02) | | 60 (0.42) | | 47 (0.74) | | 86 (0.33) | |
| **Item 5** | 123.5 (0.21) | | 70 (0.06) | | 160.5 (0.09) | | 176 (<0.001) | | 138 (<0.01) | | 236 (<0.001) | |
| **Item 6** | 80 (0.51) | | 71.5 (0.05) | | 91 (0.01) | | 211 (<0.001) | | 201 (<0.001) | | 278 (<0.0001) | |
| **Item 7** | 69 (0.61) | | 81 (0.23) | | 120 (0.58) | | 118.5 (0.15) | | 114.5 (0.44)† | | 107 (0.14) | |
| **Item 8** | 71 (0.52) | | 113.5 (0.44) | | 102 (0.92) | | 55.5 (0.81) | | 129 (0.34) | | 93.5 (0.39) | |
| **SGRQ** |  |  |  |  |  |  |  |  |  |  |  |  |
| **Total** | 154 (0.83) | | 167 (0.64) | | 178 (0.58) | | 214 (0.56) | | 176.5 (0.55) | | 172.5 (0.95) | |
| **Symptom component** | 66 (0.09) | | 83 (0.42)† | | 149 (0.73)† | | 112.5 (0.66) | | 184 (0.34)† | | 167.5 (0.63)† | |
| **Activity component** | 7 (0.53) | | 26.5 (0.68) | | 17 (0.55) | | 50.5 (0.39) | | 36.5 (0.55) | | 25 (0.84) | |
| **Impact component** | 22.5 (0.57) | | 15 (0.72) | | 16 (0.48) | | 31.5 (0.93) | | 18 (0.2) | | 23 (0.68) | |
| **mMRC** | 22.5 (0.53) | | 51 (0.33) | | 16.5 (0.23) | | 64 (0.84) | | 60.5 (0.69) | | 72.5 (0.86) | |
| **VAS for dyspnea** | 296.5 (0.55)† | | 264.5 (0.31) | | 318 (0.08)† | | 395.5 (<0.001)† | | 362 (0.01)† | | 430.5 (<0.0001)† | |

†, Satisfaction of normality in the data distribution.

SD, standard deviation; 6MWT, 6 minutes walking test; CAT, chronic obstructive pulmonary disease assessment test; SGRQ, St. George’s respiratory questionnaire; mMRC, modified Medical Research Council; VAS, visual analogue scale.

Table S 3 Reports of adverse events during the treatment period

| No. | Age | Sex | Herbal medicine | Adverse events | Time of dropout | Severity | Causality | Prognosis |
| --- | --- | --- | --- | --- | --- | --- | --- | --- |
| 1 | 80 | Male | CSBHT | Headache | Before Visit 2 | Grade 1 | Probable/Likely | Resolved after discontinuation |
| 2 | 74 | Male | CSBHT | Dyspepsia | Before Visit 2 | Grade 1 | Probable/Likely | Resolved after discontinuation |
| 3 | 79 | Male | CSBHT | Dyspepsia | Before Visit 2 | Grade 1 | Probable/Likely | Resolved after discontinuation |

CSBHT, Cheongsangboha-tang.

Figure S 1 Changes in the density of data distribution in clinical outcomes from Visit 1 (blue) to Visit 7 (red). The density of clinical outcome data distribution at Visit 1 was visualized separately for data from all patients included in the baseline regardless of dropout status (total subjects) and for data from patients excluding those who dropped out (adhered subjects), presented in that order for each outcome.

6MWT, 6 minutes walking test; CAT, chronic obstructive pulmonary disease assessment test; mMRC, modified Medical Research Council; SGRQ, St. George’s respiratory questionnaire; VAS, visual analogue scale.


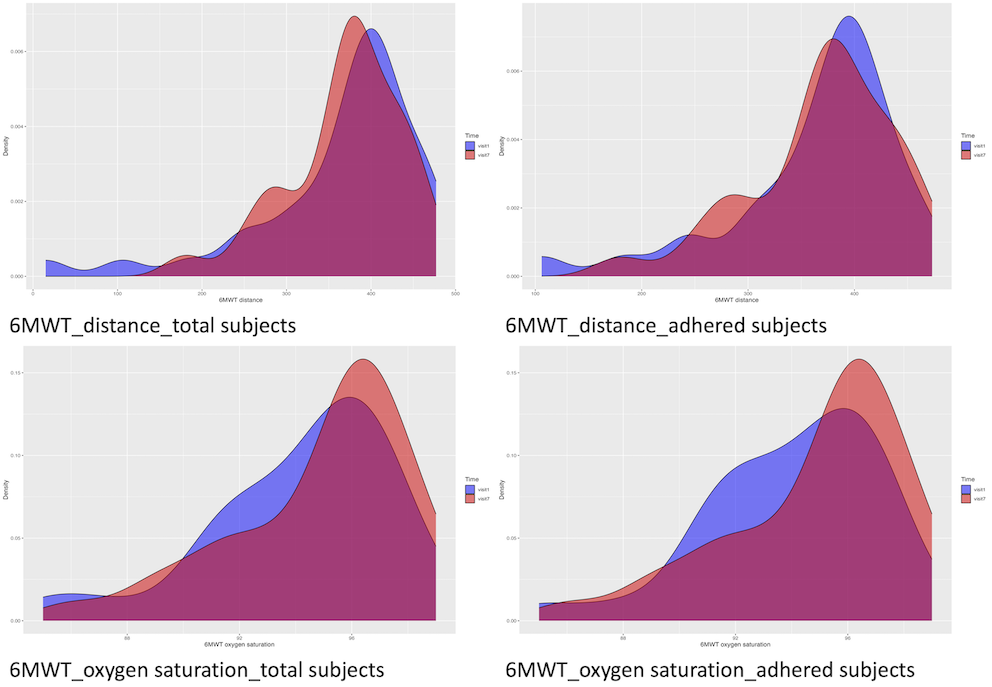

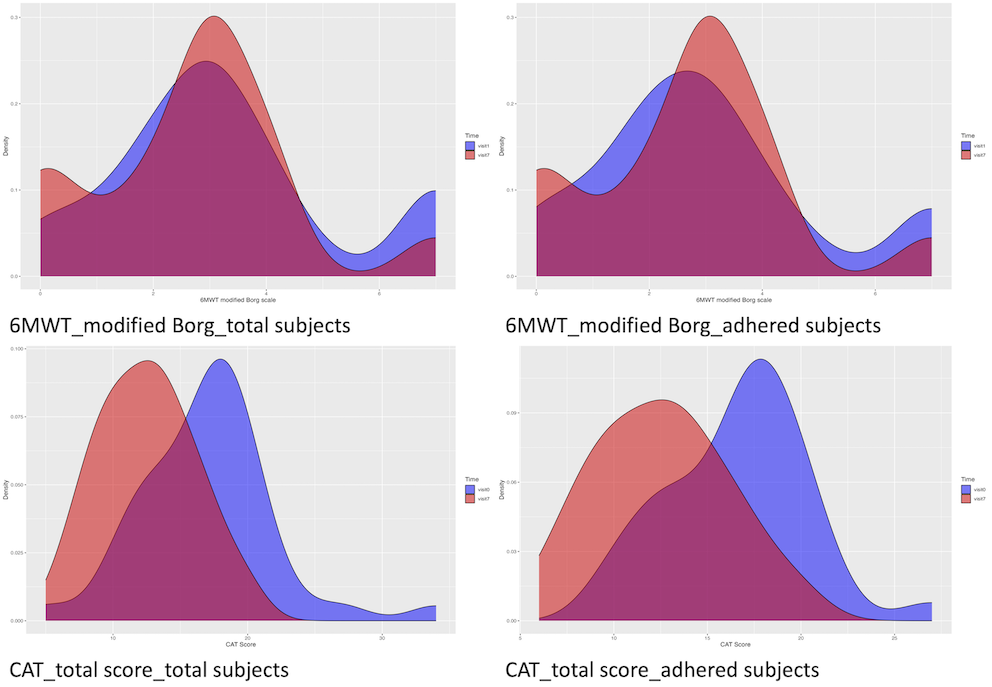

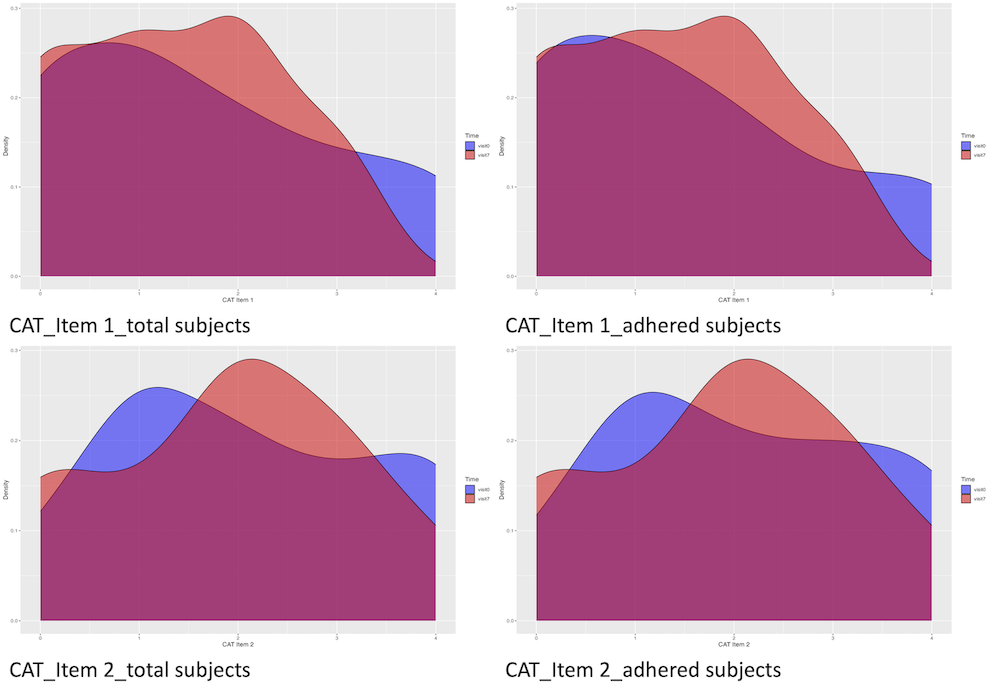

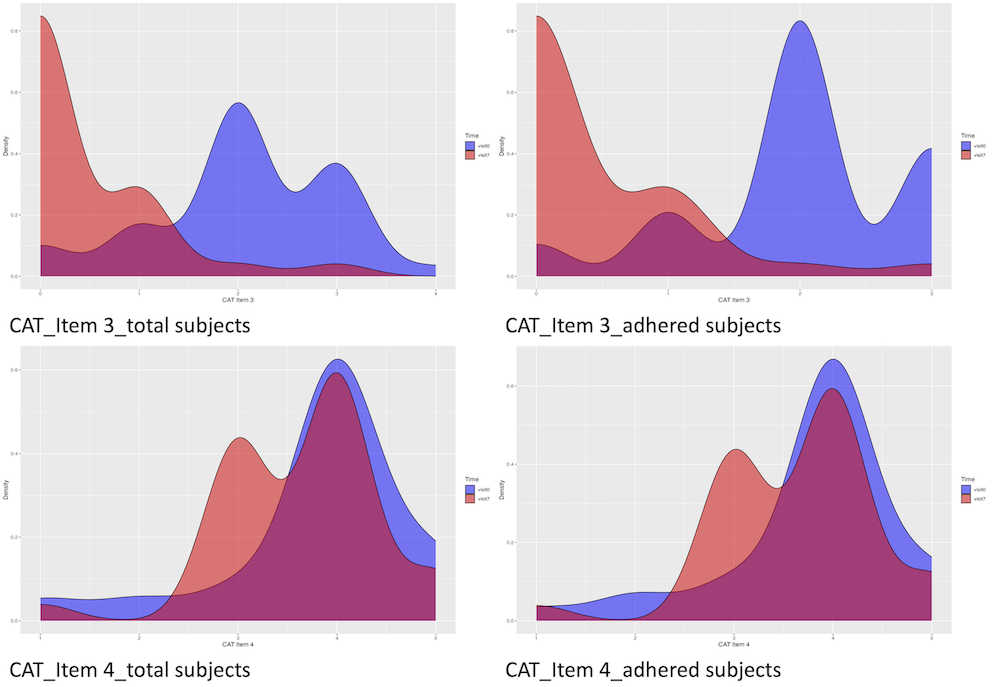

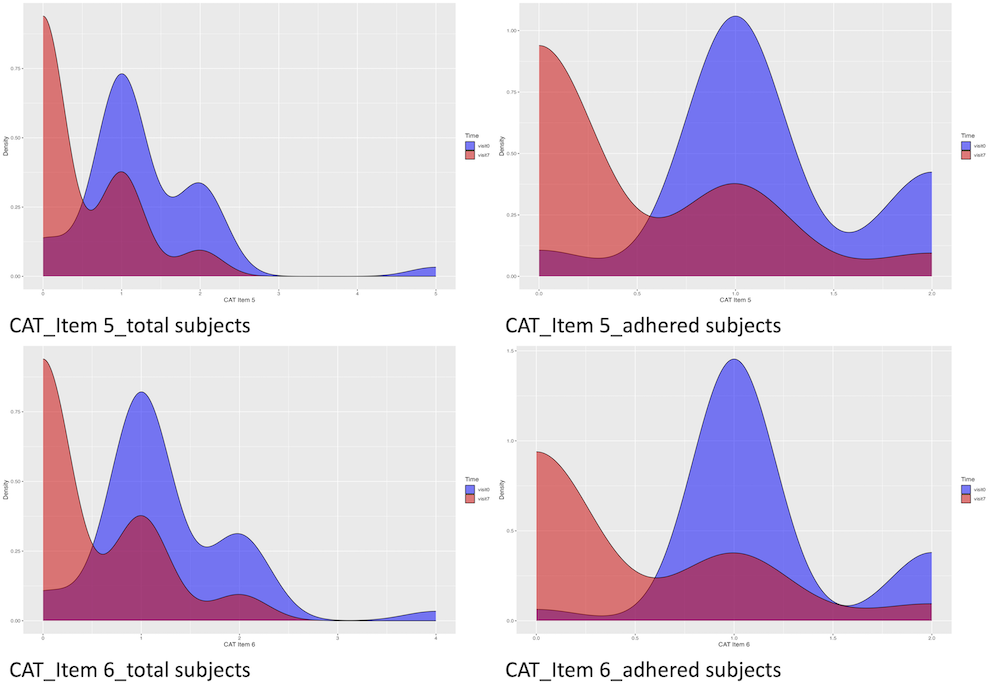

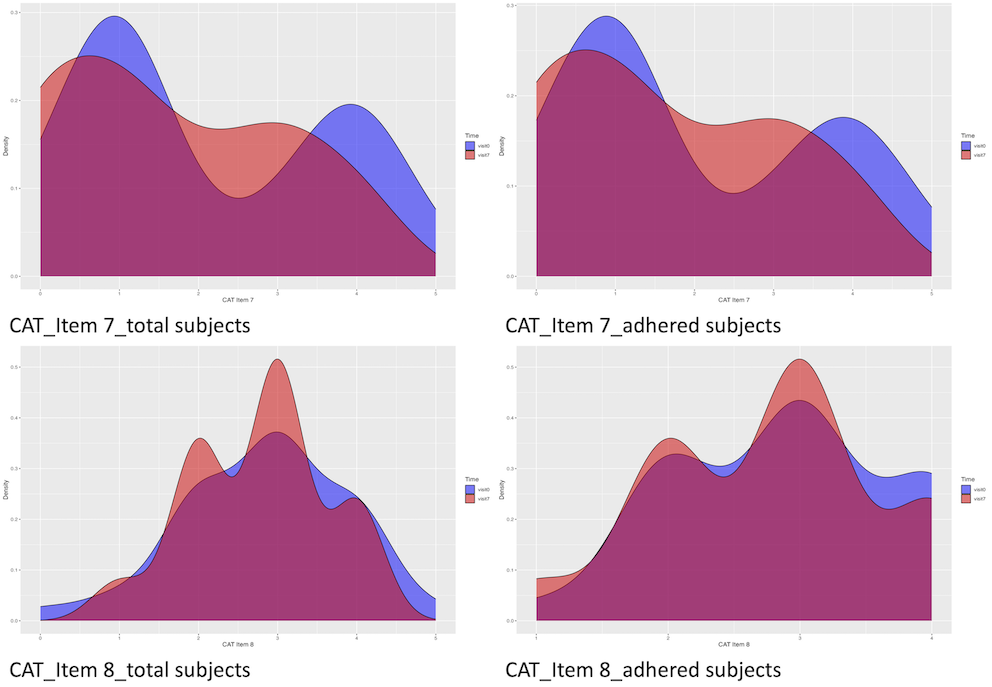

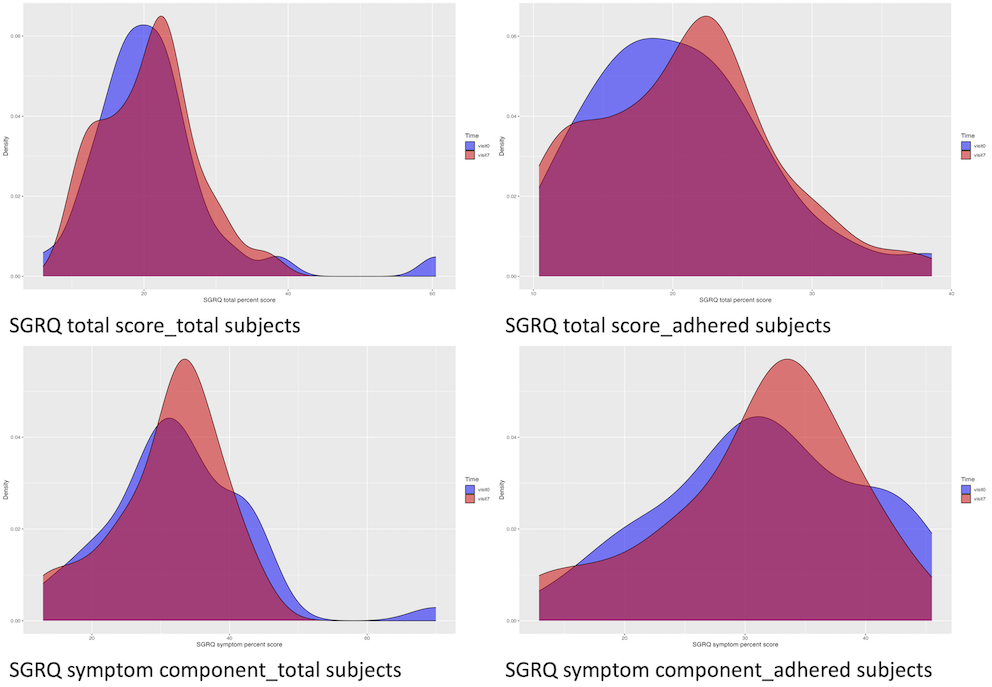

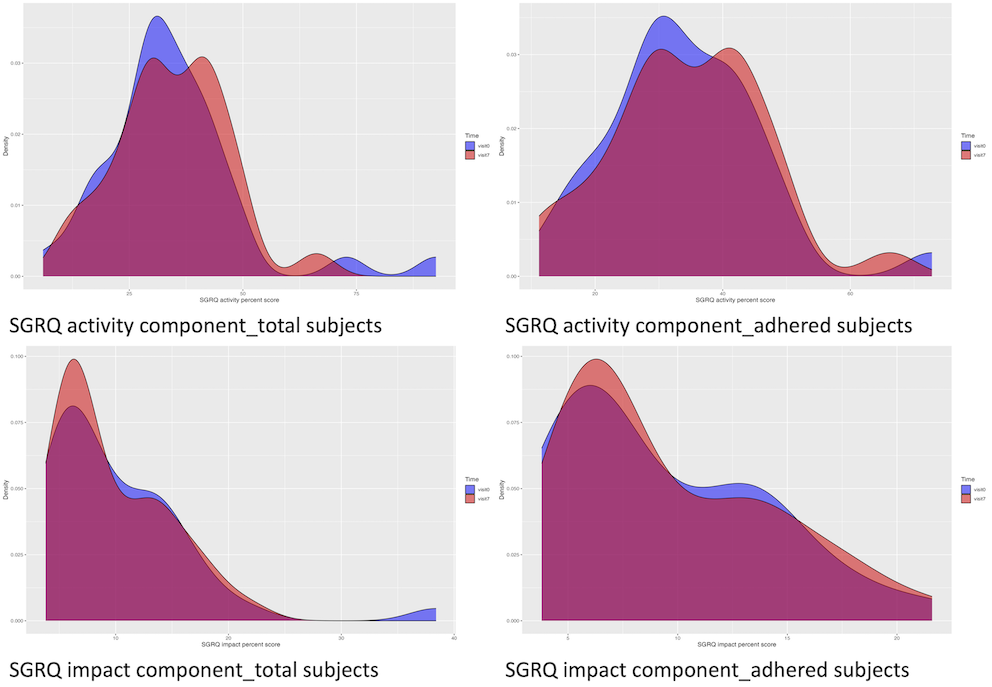

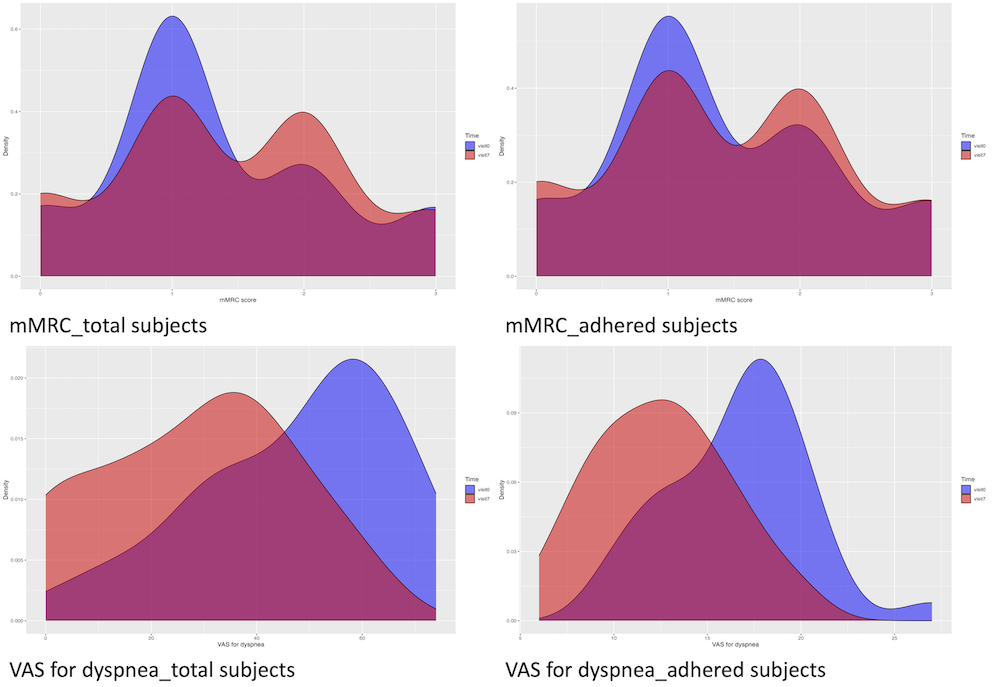


References

1. ATS Committee on Proficiency Standards for Clinical Pulmonary Function Laboratories. ATS statement: guidelines for the six-minute walk test. *Am J Respir Crit Care Med*. 2002;166(1):111-117. doi:10.1164/ajrccm.166.1.at1102

2. Puhan MA, Chandra D, Mosenifar Z, et al. The minimal important difference of exercise tests in severe COPD. *Eur Respir J*. 2011;37(4):784-790. doi:10.1183/09031936.00063810

3. Graham BL, Steenbruggen I, Miller MR, et al. Standardization of Spirometry 2019 Update. An Official American Thoracic Society and European Respiratory Society Technical Statement. *Am J Respir Crit Care Med*. 2019;200(8):e70-e88. doi:10.1164/rccm.201908-1590ST

4. Donohue JF. Minimal clinically important differences in COPD lung function. *COPD*. 2005;2(1):111-124. doi:10.1081/copd-200053377

5. Jones PW, Harding G, Berry P, Wiklund I, Chen WH, Kline Leidy N. Development and first validation of the COPD Assessment Test. *Eur Respir J*. 2009;34(3):648-654. doi:10.1183/09031936.00102509

6. Kon SSC, Canavan JL, Jones SE, et al. Minimum clinically important difference for the COPD Assessment Test: a prospective analysis. *Lancet Respir Med*. 2014;2(3):195-203. doi:10.1016/S2213-2600(14)70001-3

7. Lee S, Lee JS, Song JW, et al. Validation of the Korean Version of Chronic Obstructive Pulmonary Disease Assessment Test (CAT) and Dyspnea-12 Questionnaire. *Tuberculosis and Respiratory Diseases*. 2010;69(3):171-176. Accessed October 27, 2023. https://e-trd.org/journal/view.php?number=747

8. Jones PW, Quirk FH, Baveystock CM, Littlejohns P. A self-complete measure of health status for chronic airflow limitation. The St. George’s Respiratory Questionnaire. *Am Rev Respir Dis*. 1992;145(6):1321-1327. doi:10.1164/ajrccm/145.6.1321

9. Jones PW. St. George’s Respiratory Questionnaire: MCID. *COPD*. 2005;2(1):75-79. doi:10.1081/copd-200050513

10. Global Initiative for Chronic Obstructive Lung Disease - GOLD. Global strategy for prevention, diagnosis and management of COPD: 2020 report. Bethesda. Global Initiative for Chronic Obstructive Lung Disease - GOLD. Accessed October 31, 2023. https://goldcopd.org/

11. Nishimura K, Izumi T, Tsukino M, Oga T. Dyspnea is a better predictor of 5-year survival than airway obstruction in patients with COPD. *Chest*. 2002;121(5):1434-1440. doi:10.1378/chest.121.5.1434

12. Mador MJ, Kufel TJ. Reproducibility of visual analog scale measurements of dyspnea in patients with chronic obstructive pulmonary disease. *Am Rev Respir Dis*. 1992;146(1):82-87. doi:10.1164/ajrccm/146.1.82

13. Ries AL. Minimally clinically important difference for the UCSD Shortness of Breath Questionnaire, Borg Scale, and Visual Analog Scale. *COPD*. 2005;2(1):105-110. doi:10.1081/copd-200050655

14. Lee BJ, Jung HJ, Choi JY, Kang W, Jung SK. Preliminary Study to Develop a Korean Oriental Medical Assessment Tool for Syndrome Differentiation of Chronic Obstructive Pulmonary Disease. *Journal of Korean Medicine*. 2012;33(3):82-94. Accessed October 31, 2023. https://www.kci.go.kr/kciportal/ci/sereArticleSearch/ciSereArtiView.kci?sereArticleSearchBean.artiId=ART001702026

15. Brunson JC. ggalluvial: Alluvial Plots in “ggplot2”. R package version 0.12.5. Published online 2020. http://corybrunson.github.io/ggalluvial/.

16. CTCAE v5.0. Published 2017. Accessed October 31, 2023. https://ctep.cancer.gov/protocolDevelopment/electronic_applications/docs/CTCAE_v5_Quick_Reference_8.5x11.pdf

17. Son MK, Lee YW, Jung HY, et al. Comparison of the Naranjo and WHO-Uppsala Monitoring Centre criteria for causality assessment of adverse drug reactions. *The Korean Journal of Medicine*. 2008;74(2):181-187.

**Supplementary Table and Figure Legends**

Supplementary 1. Details of clinical outcomes and safety assessment.

Table S1. Clinical outcome changes (median and interquartile range).

Table S2. Results of the Wilcoxon signed-rank test for changes in clinical outcomes at each visit compared to Visit 1.

Table S3. Adverse events reported during the treatment period.

Figure S1. Changes in the density of data distribution for clinical outcomes from Visit 1 (blue) to Visit 7 (red).
